# Endomicroscopic AI-driven morphochemical imaging and fs-laser ablation for selective tumor identification and selective tissue removal

**DOI:** 10.1126/sciadv.ado9721

**Published:** 2024-12-11

**Authors:** Matteo Calvarese, Elena Corbetta, Jhonatan Contreras, Hyeonsoo Bae, Chenting Lai, Karl Reichwald, Tobias Meyer-Zedler, David Pertzborn, Anna Mühlig, Franziska Hoffmann, Bernhard Messerschmidt, Orlando Guntinas-Lichius, Michael Schmitt, Thomas Bocklitz, Juergen Popp

**Affiliations:** ^1^ Leibniz-Institute of Photonic Technology (IPHT), Member of Leibniz-Health-Technologies, Member of the Leibniz-Center for Photonics in Infection Research (LPI), Albert-Einstein-Str. 9, 07745 Jena, Germany.; ^2^ Institute of Physical Chemistry (IPC) and Abbe Center of Photonics (ACP), Member of the Leibniz Center for Photonics in Infection Research (LPI), Friedrich-Schiller-University Jena, Helmholtzweg 4, 07743 Jena, Germany.; ^3^ GRINTECH GmbH, Otto-Eppenstein-Str. 7, Jena, Germany.; ^4^ Department of Otorhinolaryngology, Jena University Hospital, Jena, Germany.; ^5^ Institute of Computer Science, Faculty of Mathematics, Physics & Computer Science, University Bayreuth, Universitaetsstraße 30, 95447 Bayreuth, Germany.; ^6^ Cluster of Excellence Balance of the Microverse, Friedrich Schiller University Jena, Jena, Germany.

## Abstract

The rising incidence of head and neck cancer represents a serious global health challenge, requiring more accurate diagnosis and innovative surgical approaches. Multimodal nonlinear optical microscopy, combining coherent anti-Stokes Raman scattering (CARS), two-photon excited fluorescence (TPEF), and second-harmonic generation (SHG) with deep learning–based analysis routines, offers label-free assessment of the tissue’s morphochemical composition and allows early-stage and automatic detection of disease. For clinical intraoperative application, compact devices are required. In this preclinical study, a cohort of 15 patients was examined with a newly developed rigid CARS/TPEF/SHG endomicroscope. To detect head and neck tumor from the multimodal data, deep learning–based semantic segmentation models were used. This preclinical study yields in a diagnostic sensitivity of 88% and a specificity of 96%. To combine diagnostics with therapy, machine learning–inspired image-guided selective tissue removal was used by integrating femtosecond laser ablation into the endomicroscope. This enables a powerful approach of intraoperative “seek and treat,” paving the way to advanced surgical treatment.

## Teaser

Multimodal endomicroscopy and fs-laser ablation enables AI-driven cancer detection and tissue removal.

## INTRODUCTION

The rising incidence of cancers is a major global health challenge. To fight cancer, more accurate diagnostic tools and precise function preserving surgical approaches are required. Preservation of function is most critical for cancers close to important functional structures, e.g., in the brain or close to the vocal folds in case of head and neck cancer. Head and neck cancer is among the most common types of cancer in the world, accounting for more than 850,000 new cases and 400,000 deaths annually in 2020 (*1*). Moreover, data indicate a general rising trend in incidence and mortality both in well-developed and developing countries and predict a 30% increase in annual incidence by 2030 (*2*). Timely and effective diagnosis of head and neck cancer is thus essential in the mitigation of the burden on both society and economy.

However, the accurate diagnosis of malignancies in general and specifically head and neck cancer is intricate, requiring a multimodal imaging approach combining, e.g., ultrasound, computed tomography, magnetic resonance imaging, and endoscopy. While all these imaging techniques can visualize tumor masses and their spread preoperatively, intraoperative diagnostics still rely on magnification tools, endoscopy, and microscopy to identify tumor areas.

The current gold standard in head and neck cancer diagnosis, as for any type of cancer, is based on the histopathological analysis of tissue biopsies (*3*). The pathologist preferably works with formalin-fixed and paraffin-embedded (FFPE) tissue samples. FFPE processing provides most accurate results but is time consuming, invasive, and cannot be performed intraoperatively. Instead, a so-called frozen sections examination can be performed on native tissue sections intraoperatively. This method is, however, less accurate, which is why subsequent diagnostic confirmation using FFPE sections is still necessary. In head and neck surgery, for example, the incidence of incomplete resections (R1), i.e., the subsequent discovery of tumor cells in the incision margin, is approximately 7.5 to 10%, depending on tumor size and location. In such cases, the patient must often undergo follow-up surgery and more aggressive postoperative chemotherapy and radiotherapy, which represents an enormous burden for the patient and negatively affects the patient’s prognosis. This is why, if possible, more tissue is removed during primary surgery than would be necessary to reach the tumor margin. Even being more error prone, intraoperative frozen section analysis is not available in most hospitals due to economic reasons and a shortage of pathologists. Last, both pathological workflows on frozen and FFPE-processed sections are dependent on the pathologist’s experience and promptness. Thus, head and neck tumor surgery would highly benefit from new approaches for intraoperative pathology. Intraoperative imaging technologies that can precisely locate the tumor during surgery are needed to remove it as completely as possible, as targeted detection of malignant tissue during curative surgery is the most important prerequisite for complete tumor removal.

In recent years, a large interest has been devoted to the investigation of alternative methods for in situ, low-invasive, and real-time detection of head and neck cancer or any kind of cancer, in particular with regard to the concept of “spectral histopathology”, i.e., the use of optical and spectroscopic techniques to examine tissue suspected of malignancy with respect to its morphochemistry, i.e., morphology, but more importantly, chemical composition (*4*, *5*). In this respect, multimodal nonlinear optical microscopy, including coherent anti-Stokes Raman scattering (CARS), two-photon excited fluorescence (TPEF), and second-harmonic generation (SHG) is, among others, a promising approach to assess the tissue morphochemistry in a label-free manner (*6*–*8*). Several studies have highlighted the potential of this technique in the discrimination of different types of cancer, including breast (*9*), lung (*10*), skin (*11*), and head and neck cancer (*12*, *13*). The combination of this multimodal imaging approach with recent developments in machine and deep learning technologies have made it possible to quantitatively evaluate the generated multimodal images by translating the encoded morphochemical information to differentiate healthy from cancerous tissue (*14*–*16*), paving the way for an automatic real-time cancer diagnosis.

Toward the intraoperative application of multimodal nonlinear imaging techniques for head and neck cancer detection, recent research has focused on the development of endoscopy-based systems that could bring disease detection directly to the operating room. To date, several nonlinear endoscopic solutions have been developed, most of which are compact and flexible endomicroscopes based on scanning piezoelectric tubes (*17*–*19*) or multicore fiber bundles (*20*, *21*). They allow the implementation of multiple nonlinear imaging modalities, up to five modalities in the best case (*22*), to image biological tissues in a label-free manner with subcellular resolution providing diagnostically relevant insights on the morphochemical sample composition. Stimulated Raman scattering (SRS) may have an advantage over CARS since it is a more quantitative technique (*23*) and it does not suffer from the nonresonant background (*24*). However, its implementation in an epi-scattering mode is associated with challenges, as the SRS signal is a differential signal measured at the pump or Stokes wavelength and requires complex lock-in detection schemes. Since the optics is optimized for transmitting the laser, the laser signals in epi-direction need to be detected directly at the surface with a large photodiode (*25*). This results in a substantially larger setup. SRS detection through the objective is difficult, since the laser light is transmitted back to the laser and could be guided to the detector by optical isolators, which would not work well for scattered photons. For detecting brain tumor and head and neck cancer, rigid endoscopic probes using a galvanometer scanning mechanism have been introduced in the past years (*26*, *27*). In particular, the rigid endoscope for head and neck cancer detection combines a hitherto not achieved field of view of about 650 μm with a high lateral resolution of 1 μm (*27*).

The aforementioned endomicroscopic devices have demonstrated imaging capabilities on ex vivo human tissue samples and in one case (*17*) have also been tested in vivo on a mouse. However, to the best of our knowledge, no systematic patient study has been conducted to determine the efficacy of the endomicroscopes in identifying head and neck cancer. In this work, we present for the first time a preclinical systematic study on ex vivo samples conducted on 15 patients affected by head and neck squamous cell carcinoma at the Jena University Hospital. We used the CARS/TPEF/SHG endomicroscope shown in (*27*) in combination with innovative deep learning methods to characterize ex vivo head and neck tissue samples to automatically distinguish between different tissue types and differentiate healthy tissue from tissue that needs to be resected. This is a necessary step toward clinical translation for in vivo application and validates the effectiveness of the techniques and reliability of the instrument. The model demonstrated a high sensitivity and specificity, confirming the efficacy of the compact endomicroscopic device. A total of 20 specimens from 15 different patients were analyzed this way. The samples were preserved by freezing them in liquid nitrogen and then stored at −80°C; afterward, they were cut in thin slices with a cryotome. In addition, we demonstrated the performance of a technically improved version of the endomicroscope on a freshly excised bulk human tissue sample highlighting the great potential of this device for the translation to an in vivo clinical setting. Specifically, we reduced the tolerances of the optical probe, improved the mechanical robustness of the device, and used a laser source with higher repetition rate to reduce image acquisition time and increase image quality.

To take full advantage of this multimodal endomicroscopic imaging probe that incorporates deep learning methods for automated cancer detection, a major step forward would be the implementation of spectroscopic-guided femtosecond ablation in a “seek and treat manner” as an all-optical approach for combined disease diagnosis and therapy. Femtosecond laser ablation has already found commercial application in eye surgery, such as fs-LASIK (*28*), as it allows a maximal protection of the surrounding tissue. It is increasingly gaining interest in research across several fields such as orthopedics, dentistry, and the treatment of vocal folds (*29*–*31*). In addition, its integration into microsurgery probes has been the subject of recent studies, as it offers the potential for precise and selective image-guided ablation (*31*). The head and neck area is a very narrow space and the resection of the larger tissue quickly leads to functional deficits. We hypothesize that femtosecond laser offers precise complete tumor resection with maximal protection of functionally relevant surrounding tissue. The same holds true for definitive tumor ablation but also for surgical procedure to take biopsies to confirm or rule out a tumor diagnosis. Thus, we also implemented a femtosecond laser ablation modality into the endomicroscopic probe for CARS/SHG/TPEF image-guided selective tissue removal with subcellular resolution. In contrast to previous studies investigating the potential of nonlinear imaging (e.g., CARS and TPEF) guided laser ablation of tissue and cells (*32*, *33*), we developed a test pipeline demonstrating a real case theranostic application scenario combining multimodal nonlinear imaging with automatic image processing for the automated detection and selective removal of tissue structures of interest, illuminating the way toward improving tumor diagnosis and therapy in the future.

The first section of this article displays and discusses qualitatively multimodal nonlinear images from head and neck tissue showing that by a mere visual inspection, diagnostically morphochemical details can be retrieved. To quantitatively transform the multimodal images into diagnostically relevant information, a deep learning–based semantic segmentation is applied, showing the capability of automatically detecting head and neck cancer. In a next step, the transformation from thin sections to bulk tissue measurements is demonstrated proofing the in vivo applicability of the presented artificial intelligence (AI)–driven multimodal imaging approach. Last, in the last paragraph of “Results,” the proof-of-concept pipeline for an AI-driven image-guided selective tissue ablation is presented. The results are then discussed and further perspectives on the impact of this work are provided. The last section describes in detail the setup used for the case study, sample preparation, and data analysis pipeline, including preprocessing, deep learning model, and image quality metrics used to analyze the data.

## RESULTS

### Step 1: Derivation of morphochemical details within multimodal images of head and neck tissue sections by mere visual inspection

Multimodal nonlinear images of 30-μm-thin head and neck tissue sections were acquired via our CARS/SHG/TPEF endomicroscope. The CARS excitation wavelengths, at 796 nm and 1030 nm for pump and Stokes, respectively, are tuned to match the CH_2_ stretching vibration at 2850 cm^−1^, probing methylene-rich chemical compounds, in particular lipids and, to a lesser extent, proteins (*34*, *35*). Furthermore, the CARS signal yields information on the sample’s morphological structure because of the nonresonant four wave mixing background (*36*). The chosen excitation wavelengths also generate TPEF signals coming from a variety of autofluorophores in the tissue, such as elastin, reduced form of nicotinamide adenine dinucleotide phosphate [NAD(P)H], flavin adenine dinucleotide (FAD), keratin, and collagen (*37*–*39*). SHG of the Stokes beam around 515 nm derives from the structures of collagen fibers within the sample (*38*). More details on the laser configuration can be found in the “Cart-based endomicroscopic system” section in Materials and Methods and a comprehensive description of the detection unit is included in the Supplementary Materials of our previous work (*27*).

**Fig. 1.**
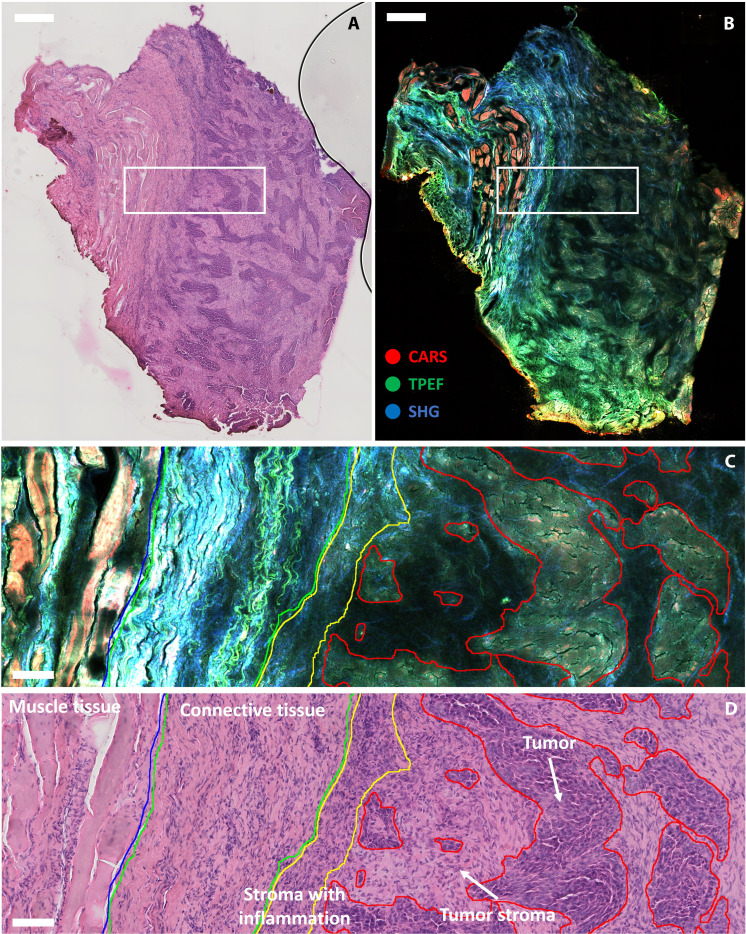
Comparison between H&E-stained and multimodal nonlinear image of a head and neck tissue section. (**A**) Large overview of a H&E-stained tissue section. Scale bar, 0.5 mm. (**B**) Large overview of the same tissue section of (A) measured with the multimodal nonlinear endomicroscope. Scale bar, 0.5 mm. (**C**) Cropped area corresponding to the rectangular selection in (B) with a line overlay of the annotations. Scale bar, 100 μm. (**D**) Cropped area corresponding to the rectangular selection in (A) with a line overlay of the annotations. Scale bar, 100 μm.

The combination of the three modalities carries thus a large amount of morphochemical information that helps in the discrimination of cancerous tissue. Figure 1 shows a qualitative comparison between the gold standard hematoxylin and eosin (H&E) staining and the merged multimodal nonlinear image acquired with the endomicroscope for one of the 20 tissue sections. A visual comparison of the cropped regions in Fig. 1 (C and D) already provides insightful distinction between the different tissue types present in the specimen. A clear delineation of connective tissue in the multimodal image in Fig. 1C is evidenced by the high level of SHG and TPEF signals from the fiber structure of collagen and elastin, major components of the connective layer (*40*). The muscle tissue on the left shows a high CARS signal within the dense muscle fibers, probably due to the high protein content. Some TPEF and SHG signal arising from intramuscular connective tissue (*41*) is also present in between muscle cells. The tumor-annotated regions drawn by the pathologist on the right side (see Fig. 1D) show a higher TPEF signal with respect to the surrounding tumor stroma (see Fig. 1C). This might be attributed to an increase in NAD(P)H concentration, which is a marker of accelerated metabolic activity in cancer cells (*42*, *43*), and has been observed to increase in the presence of cancerous regions also in previous studies (*11*–*13*). Moreover, disordered structures of collagen fibers generating SHG interconnect tumor islands within the tumor stroma.

In general, already from a qualitative assessment of the multimodal nonlinear images, a good match can be found between the tissue characteristics highlighted by CARS, TPEF, and SHG and those expressed in the H&E staining, allowing for a first indicative identification of cancerous tissue by simple visual examination.

### Step 2: Automated deep learning–based tissue diagnosis of multimodal head and neck tissue sections

For a quantitative and automated image analysis, deep learning–based approaches can aid in the differentiation of various tissue types based on multimodal images as input. Here, we used semantic segmentation to classify different tissue types within the dataset. The complete dataset comprises 23 images of 20 tissue sections originating from 15 different patients. A total of 15 different tissue classes, i.e., tissue types, which were identified by a histopathological evaluation of the H&E images by an experienced pathologist, have been considered. However, in an initial approach, some classes were merged to reduce the dimensionality to six classes, which include healthy epithelium (1% of image pixels), tumor stroma (7%), necrosis (7%), tumor (18%), other tissue (10%), and background (56%).

Our study initially implemented a leave-one-out cross-validation approach, training the model on 14 patients and evaluating its performance on the remaining patient to assess the model’s ability to generalize across unseen data. We encountered a considerable challenge associated with high patient variance, affecting the consistency of the predictions. Some folds demonstrated promising results when the validation data exhibited lower variance relative to the training data. However, others showed only partial success or poor performance, in particular in specific categories. The complexity of our dataset is further illustrated in figs. S31 and S32 of the Supplementary Materials, which include patients with diverse demographics such as age, sex, tumor localization, and subsite. To better understand the impact of biological variability on our results, we provided a detailed table in the Supplementary Materials (table S1). In addition, cross-validation results indicated that despite the limited number of training instances, the model could identify the borders of different tissue components in each sample. Nevertheless, larger regions often were incorrectly assigned to the wrong tissue class, mainly due to the limited variability among patients and the underrepresentation of some classes in the training split. This observation emphasizes the need for a training dataset with greater diversity to enhance model accuracy and robustness.

**Fig. 2.**
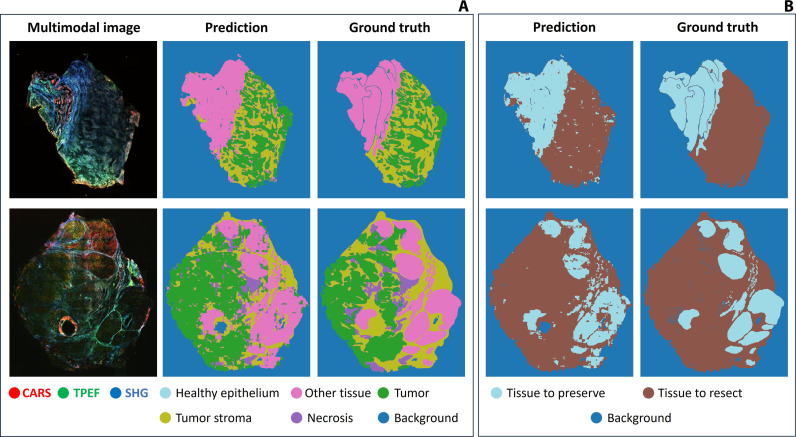
Visual assessment of CNN performance. (**A**) Six-class semantic segmentation. The left images in the panel show the original multimodal image used as input for the CNN model. The middle images represent the predictions made by the CNN. The right side includes the ground truth annotated labels from the pathologist, after regrouping. (**B**) Three classes semantic segmentation. Predictions made by the CNN (left images) and ground truth annotated labels (right).

Subsequently, to mitigate this challenge, we adopted a different approach. For training and testing, we divided the image into patches according to a chessboard pattern, selecting half of the patches for training and the others for testing. The “Deep learning model” section in Materials and Methods fully explains the dimensionality reduction and the chessboard approach, which was intended to reduce the quantity of needed training data while capturing more variability. It was found that the model could learn from the multimodal images and differentiate between various tissue types within the dataset, predicting images with high similarity to the ground truth labels from the pathological annotations. Figure 2A illustrates the prediction capabilities of the model in the reduced six-class configuration for two different samples as an example.

Table 1 reveals the model’s high specificity, especially in identifying “healthy epithelium” and “necrosis.” However, its sensitivity varies, struggling notably with “tumor stroma” and “healthy epithelium” due to their low prevalence in the dataset. This underrepresentation leads to less reliable detection of these tissues. While precision is generally robust, “tumor” and “tumor stroma” show lower reliability. The F1 score fluctuates, with the model performing well on “background,” being able to correctly detect the samples from the background signal, but less on “tumor stroma.”

**Table 1.** Performance metrics of tissue classification model in histopathological imaging evaluating specificity, sensitivity, precision, and F1 score across different tissue types with dataset configuration 1.

Class	Specificity	Sensitivity	Precision	F1 score
Background	0.97	0.98	0.97	0.97
Tumor*	0.94	0.88	0.77	0.82
Necrosis	0.99	0.82	0.92	0.87
Other tissue	0.97	0.81	0.80	0.81
Tumor stroma	0.98	0.55	0.71	0.62
Healthy epithelium	1.00	0.63	0.82	0.71
Average	0.98	0.78	0.83	0.80

* Regions marked as “dysplasia” were included in the “tumor” class, due to their low occurrence in the dataset.

Despite these issues, the model’s average performance is solid. However, enhancing sensitivity for underrepresented tissues is important, in particular in medical diagnostics, where higher precision and sensitivity is crucial for the identification of true positives for diseased regions.

To improve the model performance, we developed a second approach (Fig. 2B) consisting in the recombination of the annotation in three classes, where “tumor,” “necrosis,” and “tumor stroma” regions of the tissue are grouped in a “tissue to resect” class, and the rest of the tissue types fall into the category “tissue to preserve.” The “background” is also a separate class. This approach aims to the simple identification of malignant tissue that is considered at risk and that should be surgically removed, which is the main goal of a frozen section histopathological evaluation and the main clinical question during surgery to guide disease treatment through surgical procedure.

The classification results of this second configuration “tissue to resect” versus “tissue to preserve,” reported in Table 2, show a more balanced and effective segmentation, leading to improved metrics for “tissue to resect,” which reaches a sensitivity of as high as 90%. This restructuring, while beneficial for a correct identification of the malignant regions, still leaves some room for improvement in “tissue to preserve.”

**Table 2.** Performance metrics of tissue classification model in histopathological imaging evaluating specificity, sensitivity, precision, and F1 score across different tissue types with dataset configuration 2.

Class	Specificity	Sensitivity	Precision	F1 score
Background	0.97	0.97	0.97	0.97
Tissue to resect	0.95	0.90	0.91	0.90
Tissue to preserve	0.96	0.78	0.77	0.78
Average	0.96	0.88	0.88	0.88

### Step 3: Instrument validation and application to bulk human tissue for proofing in vivo applicability of multimodal imaging

**Fig. 3.**
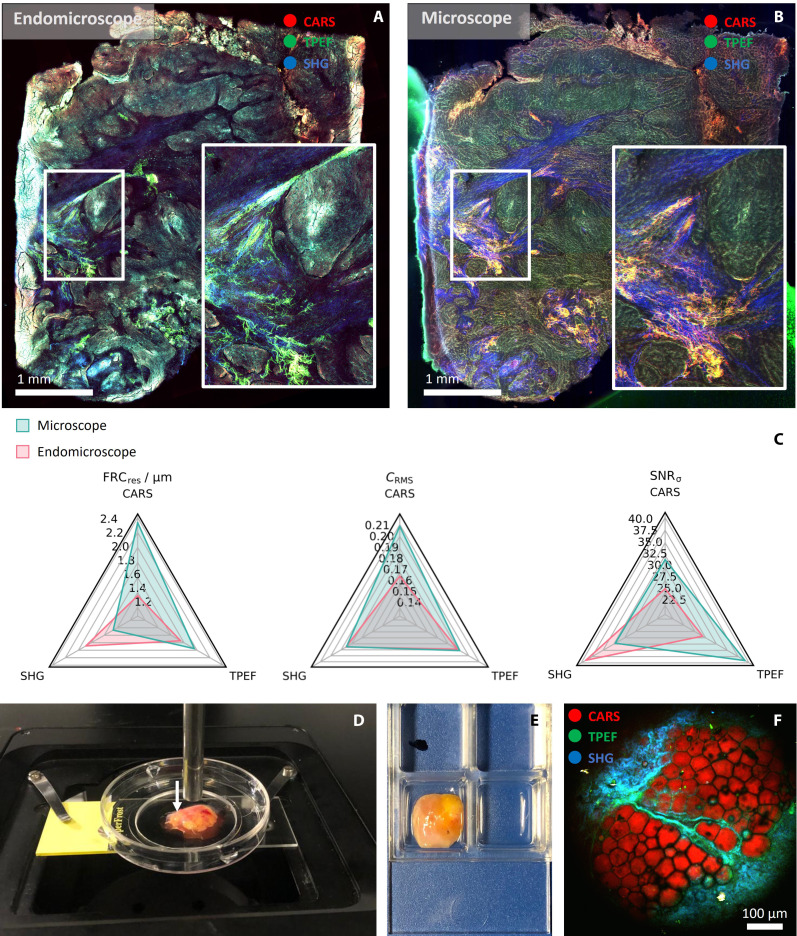
Comparison between endomicroscope and microscope and bulk tissue measurement scenario. Overview images acquired with the endomicroscope (**A**) and the commercial laser scanning microscope (**B**) on two parallel sections. The acquisition parameters were set to about 430 μm × 430 μm FOV and 1200 × 1200 pixels per tile, 3 μs pixel dwell time and 5 frames averaging in both cases. The detector settings and the laser parameters are described in Materials and Method. The zoomed insets in (A) and (B) show a zoomed area of the highlighted region of the sample. (**C**) Comparison between quality metrics computed for the two images: resolution estimated by Fourier ring correlation (FRC_res_), root mean square contrast (*C*_RMS_) and signal–to–background noise ratio (SNR_σ_); in each spider plot, the metrics computed for the two instruments are compared independently for each multimodal channel. (**D**) Photo of a bulk tissue measurement. When measuring bulk tissue, the probe is placed in soft contact with the sample to find the best focus. (**E**) Photo of the lymph node in a well plate. (**F**) Full FOV multimodal nonlinear image of the lymph node depicted in (D). The image was acquired using a second prototype of the endomicroscopic system, with same optical design. The acquisition parameters are: 2000 × 2000 pixels, 2 μs pixel dwell time, 5 frames averaging, unidirectional scanning. The sample was measured with a similar laser, a FOPG (Active Fiber Systems GmbH) at 10 MHz, with 42.5-mW pump, and 135-mW Stokes power on the sample. CARS at 2961 cm^−1^ is in red, TPEF is in green, and SHG is in blue.

To compare the imaging performance of the endomicroscope with respect to commercially available bench top microscopes, we have measured a parallel tissue section of one of the samples using a commercial confocal laser scanning microscope (SP8 Falcon, Leica Microsystems GmbH) equipped with an ultrafast laser source for coherent Raman and multimodal nonlinear microscopy (deltaEmerald, APE GmbH). Nonlinear images with similar excitation and detection scheme as used for the endomicroscopic probe have been recorded. In Fig. 3 (A and B), images acquired with the endomicroscope and the commercial microscope are compared. As for the endomicroscope, each tile was acquired with 1200 × 1200 pixels, for a 3 μs pixel dwell time and 5 frames averaging. Similar parameters were chosen for the microscope [1200 × 1200 pixels and 3.28 μs pixel dwell time over the same field of view (FOV), with 5 frame averages]. More information on the parameters can be found in the “Cart-based endomicroscopic system” section in Materials and Methods. As can be seen in the zoom insets in Fig. 3 (A and B), the endomicroscope is able to provide images of comparable quality to the bench microscope. Deviations in the features and signal strength contributions between the two images may be attributed to a variety of factors. Among them, the tissue slices measured with the endomicroscope and microscope are two 30-μm parallel sections, each one acquired with different focusing conditions; therefore, the tissue features in some regions of the images differ; the numerical aperture (NA) of the objective was slightly different, the laser excitation for the microscope featured higher spectral resolution, shorter pulse duration, and higher repetition rate, all factors that contribute to a higher signal generation and better contrast; the detectors in the commercial microscope scheme are of higher quality with respect to the photomultiplier tubes (PMTs) used in the endomicroscope custom setup, therefore allowing for higher sensitivity in signal collection. These factors contribute to a certain extent to the different visual appearance, for instance, in the higher SHG signal in the microscope image.

Nonetheless, a quantitative comparison of the image quality of the two images, based on the three image quality metrics, shows similar results between the two instruments, as illustrated in the spider plots in Fig. 3C. The resolution, estimated through Fourier ring correlation (FRC_res_), shows a similar average value among the three channels of about 1.7 and 1.9 μm for the endoscope and microscope images, respectively, although distributed differently in the channels. This variability might be generated by the different noise levels in the two detection schemes since the calculation of the resolution strongly depends on the noise floor in the image. The root mean square contrast (*C*_RMS_) ranges between 0.16 and 0.21, with generally higher values for the image acquired by the microscope. Finally, the signal-to-noise ratio (SNR_σ_) of the two instruments is also comparable, with an average value of about 31 for the endomicroscope and 34 for the microscope. In particular, the microscope gains a higher value of SNR_σ_ for the TPEF channel, thanks to its low background signal.

As mentioned above, although an exact quantitative comparison between the two systems is not directly possible because of differences in the excitation and detection schemes, the calculation of image quality metrics provides values of the same order of magnitude, demonstrating the high image quality of multimodal images acquired with our endomicroscope compared to a state-of-the-art laser scanning microscope. To show the reproducibility of the imaging techniques with different systems, we have imaged the same tissue section with the endomicroscope and with a commercial laser scanning microscope and included it as a comparison in the Supplementary Materials (see fig. S29). In comparison, the use of shorter pulse durations allows for a more efficient signal generation for our techniques, since signal strength for all three modalities depends nonlinearly on the peak intensity, which scales with pulse duration. The higher repetition rate of the microscope system, on the other hand, allows for better signal collection, in terms of averaging the contribution of multiple pulses.

To demonstrate the potential of the endomicroscopic probe in a real in vivo application scenario, we imaged a freshly excised unprocessed lymph node. To image the human lymph node, the probe was placed in soft contact with the bulk lymph node tissue (see Fig. 3, D to F). The CARS/SHG/TPEF images highlight individual adipocytes in the CARS channel (Fig. 3F) characterizing the cortex area of the lymph node. These results provide an idea of the great potential of our device to perform nonlinear multimodal imaging in vivo.

### Step 4: Machine learning–driven image-guided selective tissue ablation

In addition to disease detection, we integrated a femtosecond laser ablation platform to the endomicroscope toward image-guided intraoperative removal of suspicious tissue. Here, we developed a proof-of-concept pipeline that includes machine learning–based image processing to guide selective removal of tissue through femtosecond laser ablation. The idea behind the workflow is to demonstrate for the first time the real-time detection and subsequent removal of selected individual cells or pathological tissue structures, which is particularly important for high-precision surgery of head and neck cancer or brain tumors aiming at the preservation of function. We applied the pipeline in a test proof-of-concept study to selectively remove cholesterol crystals of cellular size in a 50-μm-thick brain tissue sample to demonstrate the high spatial resolution of the ablation process. Cholesterol crystals are a good sample model to prove the pipeline since they are similar in size to single cells or cluster of cells (tens of micrometers), thus suitable for demonstrating the high precision of the technique, and they show a distinctive SHG signature, which facilitates their identification with simple machine learning models. Moreover, brain tissue, and brain tumor in general, could be another application for implementing femtosecond laser ablation in an endoscope, since a crucial aspect when operating on this type of tissue, similarly to head and neck cancer, is surgical precision and preservation of function. This can only be achieved with a very precise ablation technique.

**Fig. 4.**
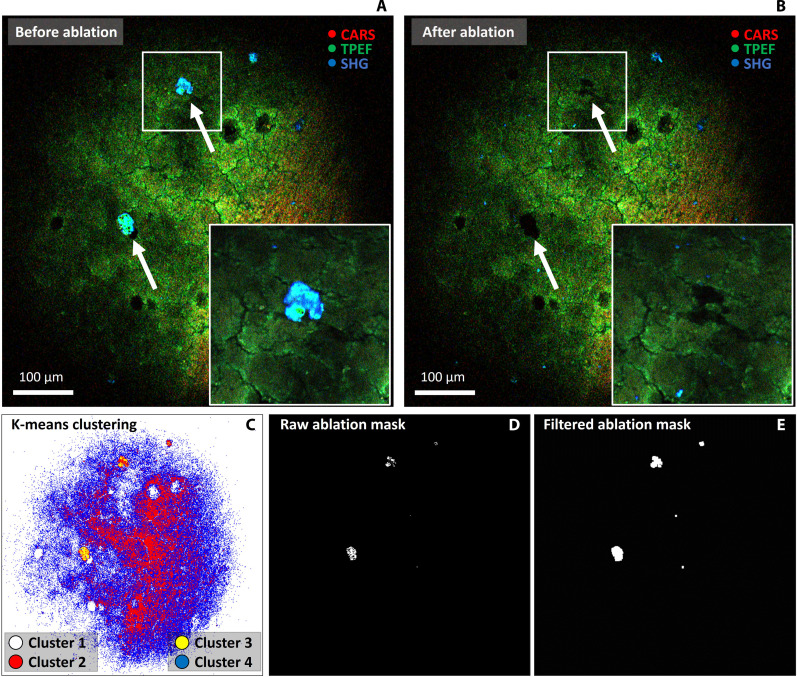
Image processing driven ablation: Proof-of-concept pipeline. (**A**) Multimodal nonlinear image of the sample before selective ablation and zoom in on a cholesterol cluster. (**B**) Multimodal nonlinear image of the sample after selective ablation and zoom in on the location of the cholesterol cluster. (**C**) K-means clustering mask (number of clusters = 4) of the acquired frame in (A). (**D**) Cluster 3 (yellow) is chosen according to a specific ablation criterion. In this case, the cluster with highest SHG intensity has been chosen. (**E**) The raw mask is spatially filtered to make it more homogeneous and remove more effectively the tissue. The filtered ablation mask is applied as input to the acousto-optic modulator of the ablation laser during scanning for 8 frames. Images are acquired with 500 × 500 pixels and 3 μs pixel dwell time. Zoom insets are cropped from a higher-resolution image of the same FOV acquired with 2000 × 2000 pixels, 1 μs pixel dwell time, and average of 5 frames.

The pipeline consists in a first acquisition of a multimodal image of the tissue with the endomicroscope, as illustrated in Fig. 4A. Cholesterol crystals are indicated with white arrows. The acquired frame is processed by applying K-means clustering to automatically divide the image into four independent regions (see Fig. 4C). Cholesterol crystals show high SHG intensity because of their crystalline nature; therefore, we decided to choose the cluster with highest SHG intensity as ablation criterion. The chosen cluster is converted into an ablation mask, as shown in Fig. 4D. The ablation mask is first filtered (see Fig. 4E), to smooth the clustering region, and then applied to the laser acousto-optic modulator to modulate the ablation laser during scanning. As a result, the targeted area is selectively ablated, as illustrated in Fig. 4B. The zoom insets in Fig. 4 (A and B) show a close-up of one of the ablated cholesterol clusters, highlighting the precision of the technique that is able to remove the crystal without damaging the surrounding tissue. Ablation for 8 consecutive frames is needed to effectively remove the crystals. A complete video that shows the real time operation scenario is included in the Supplementary Materials.

## DISCUSSION

Multimodal nonlinear CARS/TPEF/SHG imaging has proven to be an excellent tool for disease identification and has demonstrated its great potential for cancer diagnosis. For clinical translation of these advanced imaging techniques, it is necessary to develop devices for in vivo disease detection. In this regard, endoscopic probes are an ideal solution for in situ tissue characterization. Since disease-induced morphochemical changes are subtle, appropriate image analysis tools must be developed for an automated and accurate online diagnosis. In this context, deep learning approaches are a promising solution toward automatic detection of cancer.

In this study, we presented a complete endomicroscopic system implementing three nonlinear imaging modalities (CARS, TPEF, and SHG) in combination with deep learning–aided image analysis for head and neck cancer detection. We tested the system in a case study, in which we analyzed 20 tissue samples from 15 different patients. The acquired images provide insights on the tissue composition both on a morphological and molecular level and allow for a first discrimination of relevant biological components, already from a mere visual inspection. The morphochemical features observed in the endomicroscopic images correspond well with the H&E-stained images and annotations from the pathologist.

For a thorough analysis with respect to “tissue to preserve” versus “tissue to resect”, the most important question in tumor surgery, we also developed a deep learning–based semantic segmentation model. Two different configurations were considered, in which the total tissue classes were regrouped in six and three meaningful classes, respectively. The training approach consisted in the utilization of patches from each image, with the complementary patches used for testing. This allowed to increase data variability included in the training. The model shows a specificity and sensitivity of 98% and 78%, respectively, in the six-class configuration, showing only difficulties in the discrimination of tissue types that are underrepresented in the dataset, e.g., “tumor stroma.” The further rearrangement of the classes into three groups (“tissue to resect,” “tissue to preserve,” and “background”) allows for a much better performance with specificity and sensitivity values up to 96 and 88%. The latter approach aims at reproducing an actual intraoperative diagnostic application, by identifying tissue that needs resection, without the complications of multiple classes discrimination. The goal of this approach is also closer to the actual pathological question that guides the pathologist during the biopsy examination, which often comes down only to the presence or absence of malignant tissue that requires resection. The approach we developed validates for the first time the proof of concept, in a systematic 15-patient cohort study, that such imaging techniques, implemented in an endoscopic solution, can lead to automatic cancer detection.

Nonetheless, because of the limited size of the dataset, cross-validation with tests on unseen data is difficult because of the high heterogeneity of the data. A more robust and generalized approach would require a larger database of samples to capture more biological variability. This could allow the model to predict unseen images with high sensitivity and would further boost translation to in vivo studies.

The results of the image quality comparison are promising in this regard, as they show a fair similarity in the quality of multimodal images acquired by our endomicroscope compared with state-of-the-art laser scanning microscopes, where the effectiveness of nonlinear imaging techniques for cancer detection has already been proven (*9*, *13*, *15*). The instrument’s potential for in vivo measurements is also convincingly demonstrated by imaging bulk human tissue from the head and neck area, i.e., a lymph node.

In addition to disease diagnosis, the use of nonlinear multimodal imaging in combination with advanced image processing techniques has great potential for image-guided selective tissue removal. In this regard, we presented a simple workflow that integrates multimodal image acquisition and machine learning–based segmentation, in particular unsupervised K-means clustering, to guide selective femtosecond laser tissue ablation. In our example, automatic clustering helps to discriminate a region of interest and laser ablation follows by selectively illuminating that region. In principle, this approach can be generalized to any image processing method and, if combined with a deep learning model for disease detection, could serve as a tool for precise and selective head and neck cancer surgery. With this proof-of-concept pipeline, we propose a method to guide surgical intervention in the removal of diseased tissue in cases where precision is preferred over speed and the accuracy of femtosecond laser ablation might be advantageous over conventional CO_2_ lasers.

Further improvements on the technical side of the system could further increase the performance of the device and bring it even closer to clinical application. Among them, the use of a higher repetition rate and shorter pulse laser for excitation and more sensitive detectors could lead to an increase in acquisition speed while maintaining high signal quality. In addition, the use of a laser source with a higher spectral resolution, or even the implementation of broadband CARS approaches, would also enable a more chemically informative CARS channel and probe specific biomolecules with greater specificity. Moreover, further investigation of ablation products is needed to assess the safety of the technique in ablating cancerous tissues, which could generate harmful debris.

In conclusion, the presented work shows the huge potential of multimodal nonlinear imaging techniques and their implementation in an endoscopic probe, together with deep learning–based image analysis, for in situ and automated detection of head and neck cancer, facilitating the clinical workflow for more reliable and faster diagnosis and treatment in terms of selective tissue removal.

## MATERIALS AND METHODS

### Cart-based endomicroscopic system

**Fig. 5.**
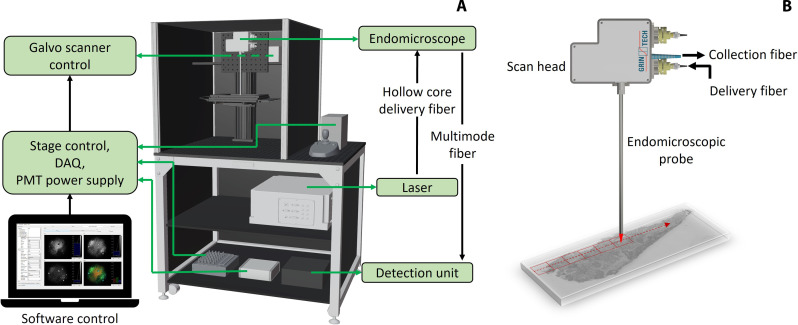
Schematic overview of the system and tile scanning scenario. (**A**) Comprehensive overview of the setup. The top section houses the endomicroscope and the galvo-scanner control. A microscope stage hosts the sample and is used to scan over the specimen, keeping the endomicroscope fixed. The middle shelf accommodates the imaging laser, which is coupled to a hollow core fiber for the delivery of the excitation light to the endomicroscope‘s scan head. On the bottom, the electronics for the control of the scanning, stage, and detectors is placed, together with the detection unit. The whole system fits a mobile rack. (**B**) Schematics of the ex vivo sample measurements. The sample is moved below the endomicroscope and several tiles are acquired, using a reduced FOV of about 430 μm × 430 μm per tile.

The system used for the case study is a complete endomicroscopic platform, fitting on a mobile cart with all its components, as depicted in the three-dimensional model in Fig. 5A. The imaging laser is a picosecond ytterbium-based fiber optic parametric generator (FOPG; Active Fiber Systems, GmbH), which emits a 30-ps pump beam at about 796 nm and a 72 ps Stokes beam at 1030 nm with a repetition rate of 5 MHz. Although the use of shorter picosecond pulses, or even femtosecond pulses, could improve nonlinear signal intensity, this would require much bulkier and more expensive laser systems, such as free-space optical parametric oscillators (OPOs), which would not be suitable for use on small medical carts. Compactness and cost are two crucial factors in endoscopic applications for clinical use; thus, a compact FOPG is preferred. The laser is delivered to the scan head of the endomicroscope via a hollow core fiber (PMC-C-R&D, GLOphotonics) and the generated nonlinear signals travel back in a large core multimode fiber (M107L02, Thorlabs) to a custom-built detection unit, where they are separated in wavelength and detected individually. The indocyanine green (ICG) fluorescence channel of the device (*27*) was not used in this study, as the samples we investigated did not contain ICG. The scan head contains the galvo-scanner (6210H, Cambridge Technology) and custom optics to couple the excitation beams to the probe head of the endomicroscope and collect back the signals to the collimation fiber. Figure 5B shows a schematic of the endomicroscope with its main components.

A DAQ acquisition card (NI 6356, National Instruments) is used to drive the galvo-scanner and the motorized stage (SCAN 75 × 50 – 1 mm, Märzhäuser Wetzlar GmbH), as well as to acquire the signals from the three photomultiplier-tubes (PMT H10723-20, Hamamatsu) used as detectors, and to regulate the voltage gain of the PMTs. The whole acquisition process is controlled by a custom-made software, based on the Scope Foundry Python framework (http://scopefoundry.org/). More details on the endomicroscope optical design, assembly, and characterization are found in our previous work (*27*).

For imaging human head and neck tissue sections, large sample overviews are needed to cover the entire area of the specimens, which can reach nearly 1 cm^2^. For this purpose, imaging was performed by tile scanning, sequentially acquiring individual tiles, and moving the stage so that a raster scan is performed during scanning, as shown in Fig. 5B. The maximum field of view of the endomicroscope is 650 μm (*27*); however, single tiles of about 430 μm × 430 μm were acquired to reduce vignetting effects at the outer borders. To take advantage of the device’s full optical resolution, each tile was captured with 1200 × 1200 pixels, resulting in a pixel size of 360 nm × 360 nm. The pixel dwell time was set to 3 μs and 5 frames were averaged per each tile. The laser power at the sample was set to about 18.5 mW for the pump and 106 mW for the Stokes, for all the tissue sections measurements. The Stokes pulse power has been increased compared to the theoretical optimal pump-Stokes ratio of 2:1 for CARS imaging to take into account the contrast of the SHG signal, which benefits from a higher Stokes intensity, and to exploit the full laser power, which inherently has a higher Stokes beam intensity.

The bulk tissue measurements of the lymph node were performed with a second prototype of the device, which had the same optical design but a more robust mechanical assembly. The second prototype used also a different laser, a picosecond ytterbium-based FOPG (Active Fiber Systems, GmbH) with a higher repetition rate of 10 MHz. In the example in Fig. 3 (D to F), the wavenumber was set to 2961 ± 25 cm^−1^ and the laser power at the sample plane was 42.5 mW for the pump and 135 mW for the Stokes.

For the image quality comparison, multimodal nonlinear images were acquired with a commercial laser scanning microscope (SP8 Falcon, Leica Microsystems GmbH). The system features an OPO-based laser system (deltaEmerald, A.P.E. GmbH), which emits a 0.85-ps tunable pump and 1.2-ps Stokes at 1032.6 nm and 80-MHz repetition rate. The pump wavelength was set to 797.5 nm, with a CARS wave number of 2855 cm^−1^. The average power was set similar to that of the endomicroscope, specifically 106 mW for the Stokes and 22 mW for the pump on the sample and a 10X/0.45-NA objective (HC PL APO 10X/0.45 dry) was used for excitation. The detection parameters were also chosen to emulate the detection unit of our cart system. CARS was acquired in the range 630 to 670 nm, SHG of the Stokes was detected between 514 and 519 nm, and the TPEF channel collected all the signal below 495 nm. For the overview image, 430 μm × 430 μm tiles composed by 1200 × 1200 pixels were acquired, with a pixel dwell time of 3.28 μs and 5 frames averaging per tile.

The second prototype of the device was also used for image processing-guided ablation. The previously described 10-MHz FOPG was used for imaging, with an average power to the sample of 43 mW for the pump and 49 mW for the Stokes. Multimodal images were acquired with a field of view of 500 × 500 pixels, on 650 μm × 650 μm, and a pixel dwell time of 3 μs. A chirped pulse amplification (CPA) ytterbium-based fiber laser (Active Fiber Systems GmbH) was used as an additional laser source for selective tissue ablation. The laser was coupled in the delivery fiber of the endomicroscope collinearly to the imaging laser. The CPA system can deliver laser pulses up to 10 μJ with a minimum duration of about 360 fs at 1032 nm, with a tunable repetition rate from 190 kHz to 19 MHz. The ablation experiments in Fig. 4 were performed with an average power at the sample of 58 mW with a repetition rate of 190 KHz, corresponding to a pulse energy of about 0.3 μJ. As observed in our previous study (*27*), the effect of linear and nonlinear dispersion in the endoscope is limited, allowing for pulse durations well below the picosecond, even for higher energies per pulse (up to 0.5 μJ). This is due to the careful design of the system, which uses a hollow-core delivery fiber, an optimized probe design where the intermediate foci are in air, rather than in glass, and a prechirping option in the laser source that allows for partial dispersion compensation.

### Sample preparation

Overall, 20 tissue samples were taken during tumor surgery from 15 patients diagnosed with head and neck squamous cell carcinoma. These samples were preserved by freezing in liquid nitrogen and then stored at −80°C until further measurements. The study was approved by the ethics committee of the University Hospital Jena (no. 4291-12/14). Written informed consent was obtained from each patient. After collection, frozen sections with a thickness of 30 μm were cut with a cryotome (Leica Biosystems GmbH, Wetzlar, Germany). First, the multimodal measurements with the endomicroscope were performed. Then, the same sections were stained with H&E. Last, an experienced pathologist annotated on each section the following histologies: healthy epithelium, necrosis, tumor, tumor stroma, dysplasia, inflammation, muscle, connective tissue, adipose tissue, blood vessels, cartilage, glandular tissue, and other. The normal lymph node used for the bulk measurements was also obtained during head and neck surgery from a patient not undergoing tumor surgery. The fresh sample was taken directly from the operating theater to the laboratory without any delays.

For femtosecond laser ablation tests, unprocessed fresh cerebral tissue from the cerebellum of *Sus scrofa domestica* has been shock frozen in liquid nitrogen and frozen sections 50 μm thickness have been prepared using a microtome (CM 3050 S, Leica, Germany).

### Data preprocessing

**Fig. 6.**
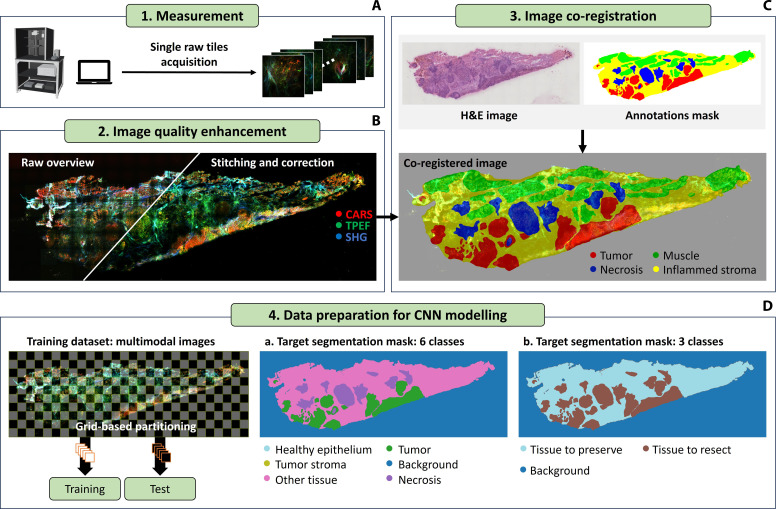
Data preprocessing pipeline. (**A**) Single raw tiles acquisition. From the acquisition software, single tiles are acquired with a spatial overlap of 10% and saved on the computer ‘s storage. (**B**) Image quality enhancement. The tiles are stitched together to create the overview image and uneven illumination artifacts that result from the illumination not being uniformly distributed throughout the field of view are corrected. (**C**) Image coregistration. The multimodal nonlinear images are coregistered with the annotations from the pathologist. The annotations are intrinsically coregistered with the H&E-stained image and saved as binary masks using the QuPath software (https://qupath.github.io/). An alignment between the multimodal image and the H&E image is then sufficient for the colocalization. The multimodal images with the associated annotations mask are then provided to the deep learning model. (**D**) Schematics of the CNN modeling data preparation. The original 15 classes are regrouped in two different subsets. In the six-class classification task they are merged into six (healthy epithelium, tumor stroma, other tissue, necrosis, tumor, and background). In a three-class classification, they are further condensed into three (background, tissue to resect, and tissue to preserve). For training and testing, the original images are divided into patches; white patches are designated for testing, the others for training.

The entire image preprocessing pipeline is depicted in Fig. 6, from the measurement acquisition to the preparation of the input images for the deep learning model. The custom acquisition software generates individual tile images acquired with 10% spatial overlap along *x* and *y*. A complete overview image is generated by removing 5% of the tile size from each border of the individual acquisitions and by stitching the tiles together. All composite images are percentile normalized, with lower percentile of 1.5% and upper percentile of 99%, to improve the dynamic range of the acquisitions, reduce the background and remove few bright pixels. Even though the tile size was chosen to be smaller than the maximum FOV to reduce vignetting artifacts, the signal still has reduced intensity at the outer edges of each tile, resulting in a visible grid pattern on the overview image. Periodic intensity fluctuations not only affect the visual appearance of the image but can also affect the results predicted by the deep learning model; therefore, a proper correction of such uneven illumination artifacts is performed. The correction is carried out using the BaSiC algorithm (*44*) through its implementation on Fiji-ImageJ (*45*). BaSiC is applied to the stack of row tiles before the removal of the overlap region, and it is executed with estimation of shading profiles and automatic selection of regularization parameters. For consistency across the prediction dataset, all the multimodal images were preprocessed and corrected for uneven illumination following the same workflow. One possible consequence of this approach is the presence of residual artifacts in a few images that might be reduced by fine-tuning the processing workflow for specific measurements. In figs. S26 to S28, we show that the presence of weak residual artifacts does not affect the underlying features identified by the model. Despite the residual uneven illumination in few BaSiC outputs that are clearly visible, the segmentation masks generated from different corrections (*46*) do not differ in a relevant way. A similar result is obtained for a direct prediction from the raw images. In this latest configuration, however, periodic patterns are generated in the segmentation results, and they can be attributed to the strong uneven illumination in the raw image. This result demonstrates that, although the algorithm provides stable results in presence of alternative correction methods, it is essential to reduce experimental artifacts for a correct image analysis.

Afterward, the corrected images are coregistered with the H&E image and the annotations mask. In most cases, fully automatic registration through a simple translation or rigid body transformation with the “MultiStackReg” plug-in (*47*) on Fiji-ImageJ was carried out to achieve a successful registration. Only in two cases, where the sample morphology had changed substantially after the H&E staining process, a semi-automatic registration was necessary. In that case, the “Align image by line ROI” plug-in (*48*) was used.

### Deep learning model

Because of the limited size of the dataset, we trained and tested the deep learning (DL) model with two different configurations of the annotated classes. Initially, we combined the 15 original classes into six meta classes: “epithelium” and “squamous epithelium” are grouped as “healthy epithelium,” representing normal tissue structures. “Tumor stroma” is maintained as a separate category. A broad category named “other tissue” is created for tissues not directly related to tumor or healthy epithelium; this includes “blood vessels,” “adipose tissue,” “stroma with inflammation,” “glandular tissue,” “connective tissue,” “muscle tissue,” and “cartilage tissue.” Last, “tumor” and “dysplasia” are classified under “tumor,” due to the low occurrence of “dysplasia” in the dataset, indicating tissues with pathological alterations. “Necrosis” remains a distinct category due to its unique pathological characteristics. As a result, the first configuration is composed of the following six classes: “healthy epithelium” (1%), “tumor stroma” (7%), “other tissue” (10%), “necrosis” (7%), “tumor” (18%), and “background” (56%).

The second segmentation approach is a further simplification of the six-class configuration. In this case, we merged “tumor,” “necrosis,” and “tumor stroma” into a single “tissue to resect category” and we renamed the other tissue class as “tissue to preserve.” This resulted in three broad classes: “background”, “tissue to resect,” and “tissue to preserve.” This simplification was necessary to refine the dataset for a more efficient modeling process.

The dataset was split into training and test sections by splitting the original composite images into patches by following a grid-based partitioning. The patches were generated by a chessboard pattern, as shown in Fig. 6D. The white patches are reserved for testing, while the other patches are allocated for training. This method aims to optimize the volume of training data and capture a diverse range of attributes, which is crucial for robust model training. A total of 498 tiles were extracted for training and an independent set of 477 tiles for testing, both from the same 23 images. In addition, 50 tiles from the training set were reserved for validation. We trained for 400 epochs and incorporated early stopping into our training protocol. This approach monitors the validation loss and halts training if there are no improvements after 20 epochs, thereby preventing overfitting. Augmentation was applied to patches of 512 × 512 pixels to generate new data during the optimization process, including perspective distortion, rotation, flip, contrast, brightness, and random cut of (226,226) pixels.

The model architecture for both configurations is based on UNet3+ (*49*), which is a specific type of convolutional neural network designed for semantic segmentation. The model processes input images of size (224, 224, and 3) and includes five down-sampling and five up-sampling stacks. The Rectified Linear Unit (ReLU) activation function is used with SoftMax on the final layer for multiclass classification.

UNet3+ integrates a VGG16 backbone with ImageNet pretrained weights, enhancing feature extraction and reducing the risk of overfitting. During training, the backbone and batch normalization layers were frozen to maintain pretrained features. The model operates with a batch size of 16 and uses Sparse Categorical Cross entropy for the loss function. It also uses the Adam optimizer with a learning rate of 1 × 10^−5^, which optimizes its performance and accuracy based on the annotated data.

The first configuration model has six outputs (“healthy epithelium,” “tumor stroma,” “other tissue,” “necrosis,” “tumor,” and “background”) and has 35.9 million parameters (21 million trainable and 14.7 million nontrainable). The second configuration recognizes three outputs (“background,” “tissue to resect,” and “tissue to preserve”), and the number of stacks is four instead of five. This model has a total of 32.2 million parameters, 17.5 million trainable, and 14.7 million nontrainable parameters. We used a tiling strategy to address grid artifacts and border issues in image analysis. Specifically, each image was processed 20 times with different tile positions, which varied slightly each time. We then used majority voting to determine the class of each pixel, aggregating the results from the multiple shifts. This technique notably minimized grid artifacts and noise, improving the quality and accuracy of our analysis. For an unbiased evaluation of our method, we used the initial output and ensured that training data did not contaminate our test sets. This approach guaranteed an accurate measure of our model’s performance.

The predicted outcomes assessed are using both qualitative and quantitative approaches. A visual analysis of the predictions and the confusion matrices given in the Supplementary Materials offer a graphical overview of how well the model has performed. Furthermore, the following metrics provide a comprehensive numerical evaluation that presents a detailed quantification of the model’s efficiency. The quantitative metrics depend on the number of true-positive (TP), false-positive (FP), and false-negative (FN) pixels, and range from 0 to 1.

1) Specificity (*S*), or true-negative rate, measures the proportion of correctly identified true negatives and it is defined as S=TN/(TN+FP), adding depth to understanding the model’s performance in recognizing negative cases.

2) Sensitivity or recall (*R*), assesses the ability to identify all relevant instances. Defined as R=TP/(TP+FN).

3) Precision (*P*), measures the accuracy of positive predictions. Calculated as P=TP/(TP+FP).

4) *F1* score, harmonizes precision and recall, providing a balance between them. Expressed as F1=2PR/(P+R).

The segmentation metrics are computed for each class and then averaged with equal importance to each class.

### Quality metrics for image evaluation

We selected established image metrics to assess the performance of the endomicroscope, compared to a commercial laser scanning microscope. The metrics are computed to demonstrate that the endomicroscopic and microscopic acquisitions are characterized by comparable parameters.

1) Image resolution. The resolution is estimated by the FRC, a method validated for optical microscopy applications to evaluate the maximum spatial frequency with relevant spectral SNR (*50*). We implemented single-image FRC (*51*), and set a threshold of 1/7 to detect the maximum spatial frequency. The resolution is then estimated as the inverse of the spatial frequency, rescaled according to the number of pixels of the image, and converted in micrometers. The FRC curve is smoothed by an average filter of 13-pixel size to remove sharp peaks that may affect the resolution estimation.

2) Root mean square contrast. The root mean square contrast is defined as the SD of the pixel intensities of the image, calculated over all the pixels *s_i_* of an image *s* composed of *N* pixels. $\mu_{s}$ is defined as the average image intensity

$$C_{RMS}=\sqrt{\frac{1}{N}\sum_{i}{\left(s_{i}-\mu_{s}\right)}^{2}}$$ (1)

3) SNR. We estimate the ratio between the signal range of the measurement and the background noise of the image, defining the SNR as

$${\text{SNR}}_{\sigma }=20{\text{log}}_{10}\frac{s_{\text{max}}-\mu_{\text{bg}}}{\sigma_{\text{bg}}}$$ (2)

Where $s_{\text{max}}$ is the maximum value of the image, and $\mu_{\text{bg}}$ and $\sigma_{\text{bg}}$ are the average value and the standard deviation (SD) of a selected background region, respectively.
