# Supplementary material for: Endomicroscopic AI-driven morphochemical imaging and fs-laser ablation for selective tumor identification and selective tissue removal
Source: Sci Adv. 2024 Dec 11;10(50):eado9721. doi: 10.1126/sciadv.ado9721 (PMC11633757; doi:10.1126/sciadv.ado9721)
Supplement: Supplementary file 1 — Supplementary Text Figs. S1 to S32 Table S1 Legend for movie S1 [file sciadv.ado9721_sm.pdf]

Science Advances
AAAS

Supplementary Materials for

Endomicroscopic AI-driven morphochemical imaging and fs-laser ablation for selective tumor identification and selective tissue removal

Matteo Calvarese *et al.*

Corresponding author: Juergen Popp, juergen.popp@uni-jena.de

Sci. Adv. **10**, eado9721 (2024)
DOI: 10.1126/sciadv.ado9721

The PDF file includes:

Supplementary Text
Figs. S1 to S32
Table S1
Legend for movie S1

Other Supplementary Material for this manuscript includes the following:

Movie S1

Supplementary Text

Complete setup installed at the Jena University Hospital

The cart-based endomicroscopic system described in the manuscript has been mounted on a mobile cart and installed at the Jena University Hospital, where the measurements for the case study were performed. Fig. S1 shows an actual photo of the complete system.

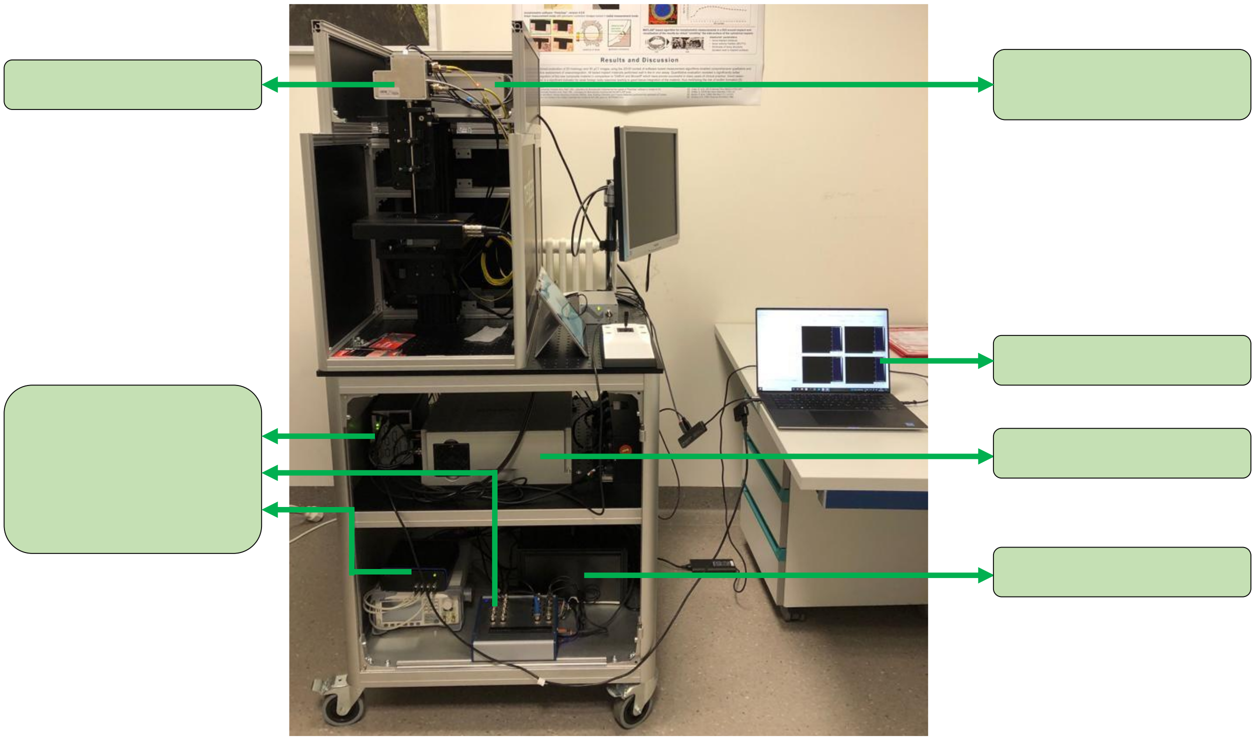

Fig. S1.

Photo of the complete setup installed at the Jena University Hospital for *ex vivo* tissue measurements. Green boxes illustrate the main components of the system.

Supplementary Text

Extended visualization of the CNN model results

The following figures show the comparison between CNN model prediction and pathologist ground truth for the two different approaches (6-class and 3-class semantic segmentation) for the full dataset. The dataset consists of 23 images from 20 samples. Three samples were divided into two images to reduce the measurement time.

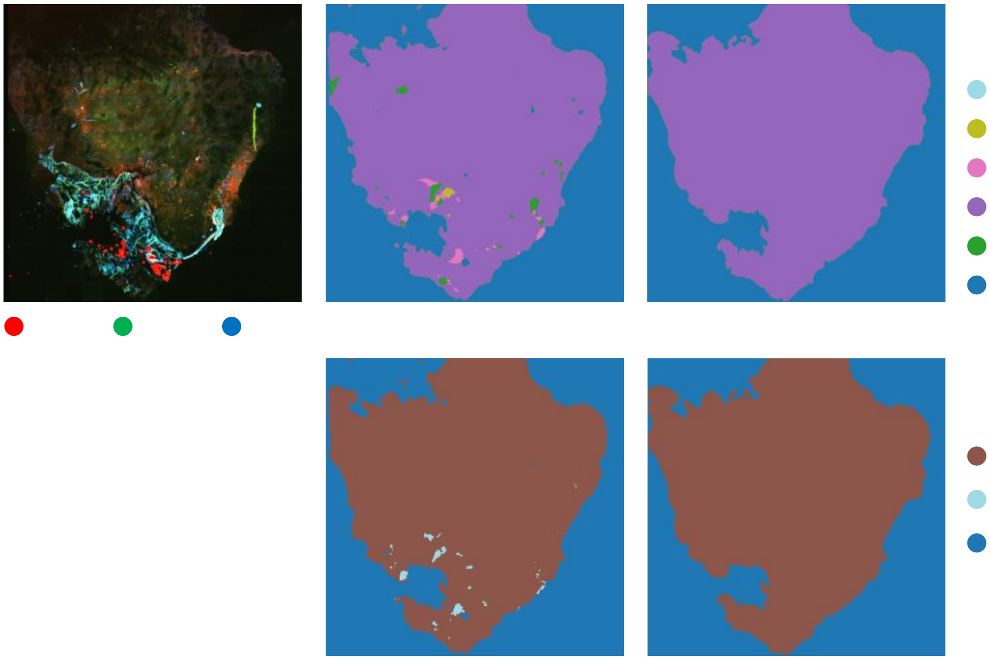

Fig. S2.
Model performance for sample 01.

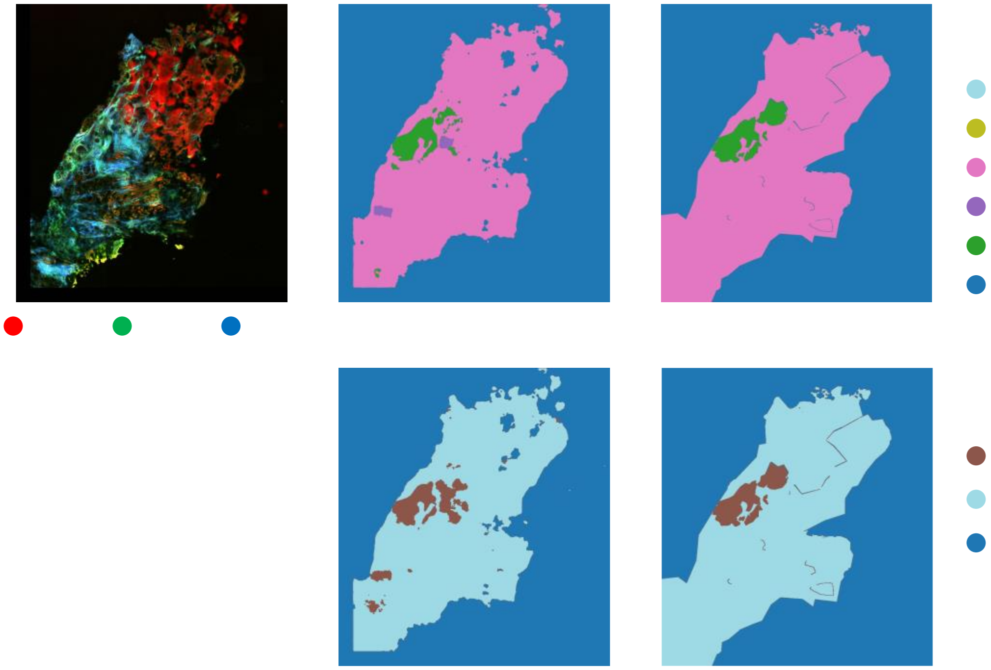

Fig. S3.

Model performance for sample 02-part1.

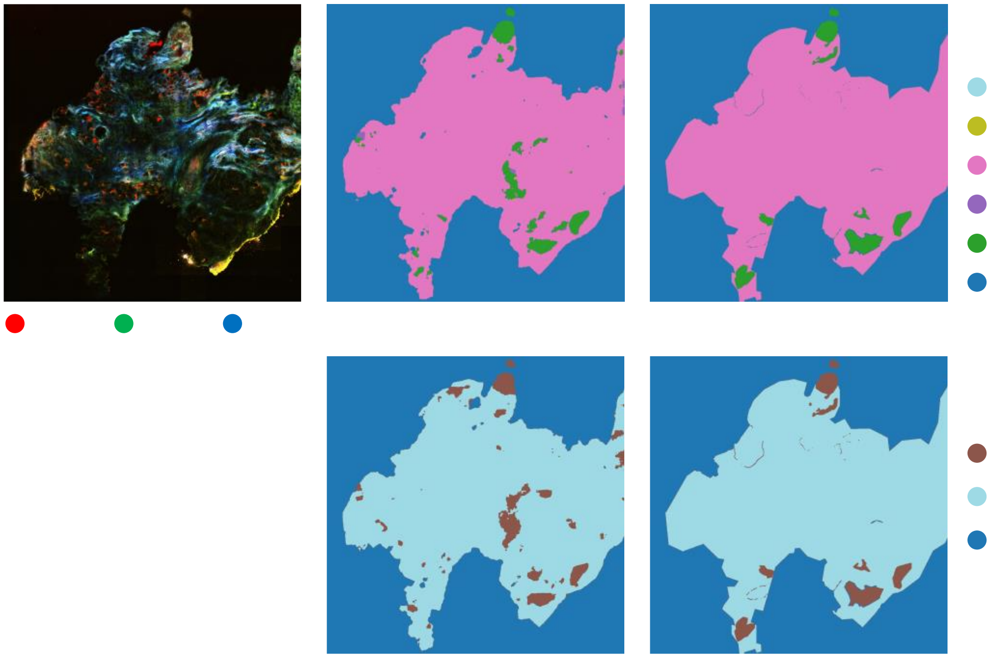

Fig. S4.

Model performance for sample 02-part2.

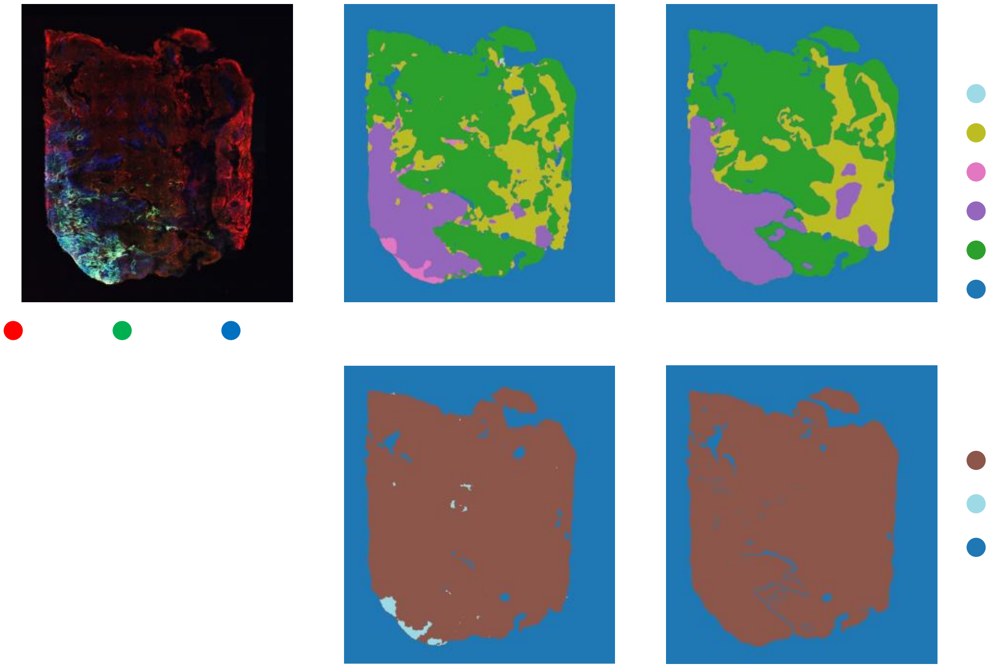

Fig. S5.

Model performance for sample 03.

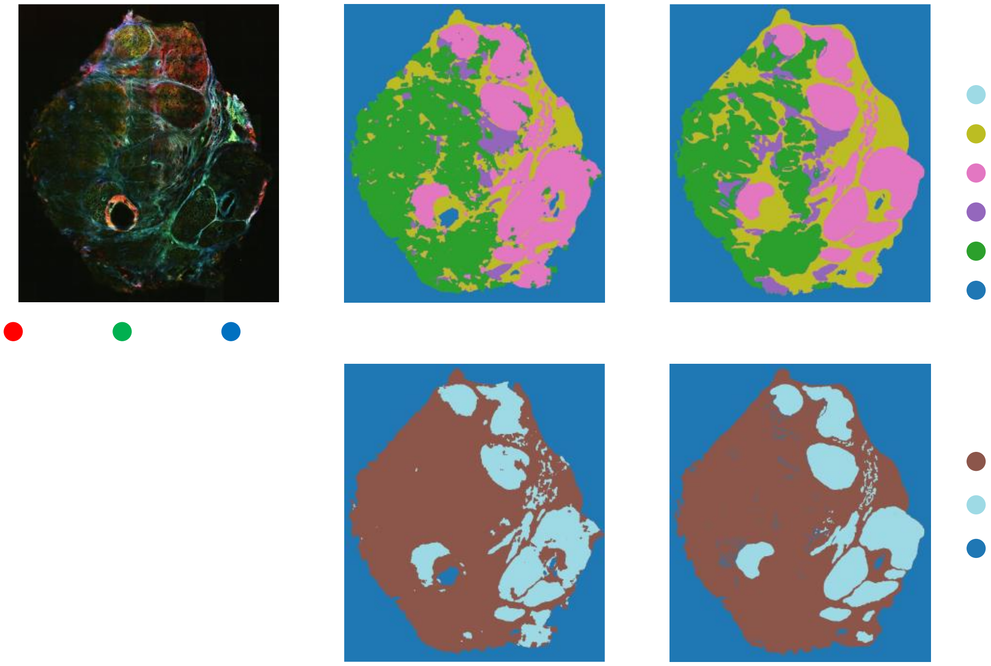

Fig. S6.

Model performance for sample 04.

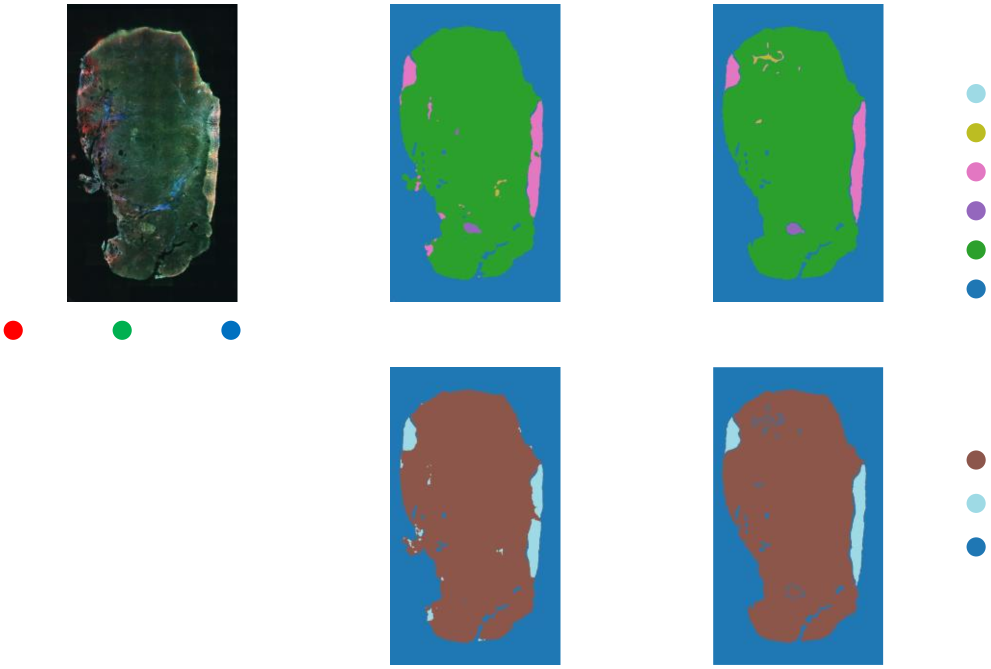

Fig. S7.

Model performance for sample 05.

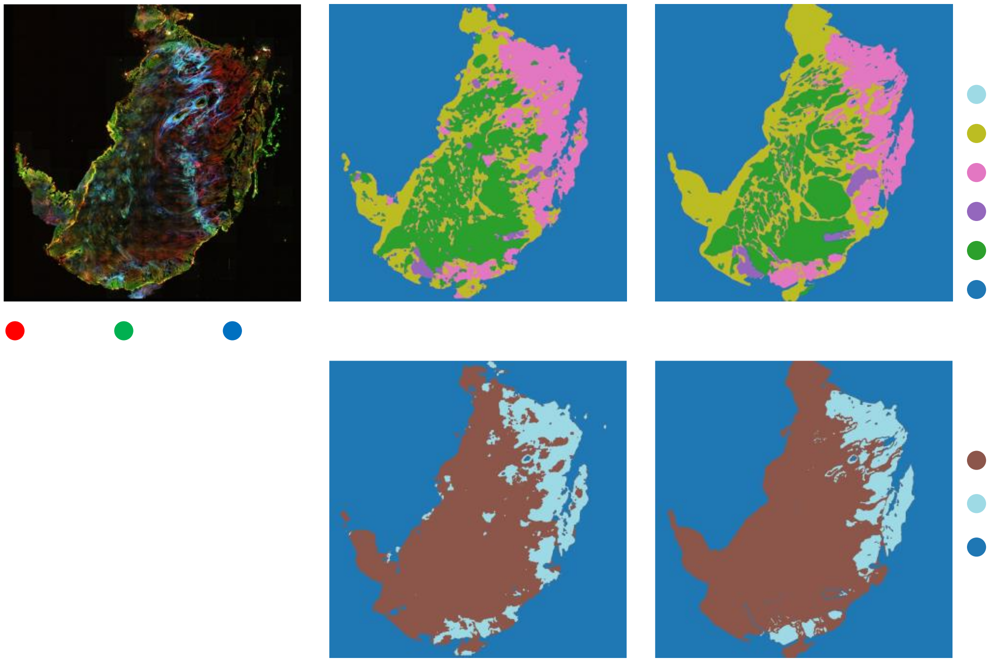

Fig. S8.

Model performance for sample 06.

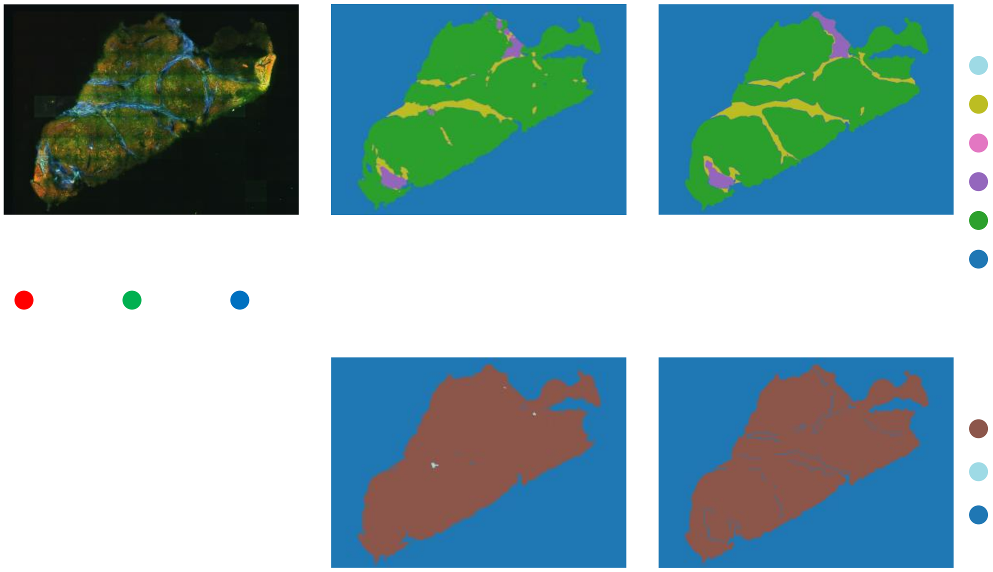

Fig. S9.
Model performance for sample 07.

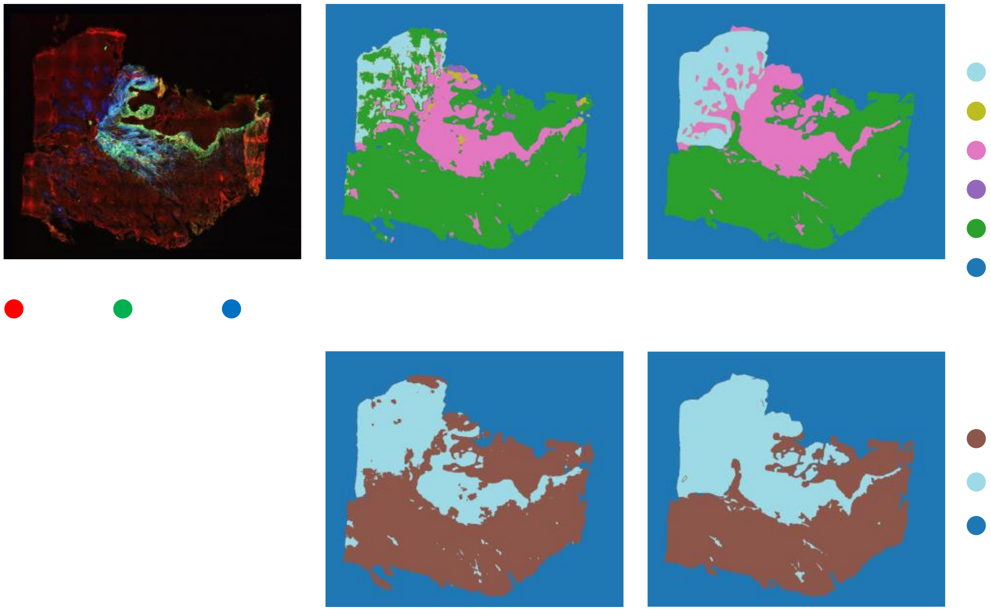

Fig. S10.
Model performance for sample 08.

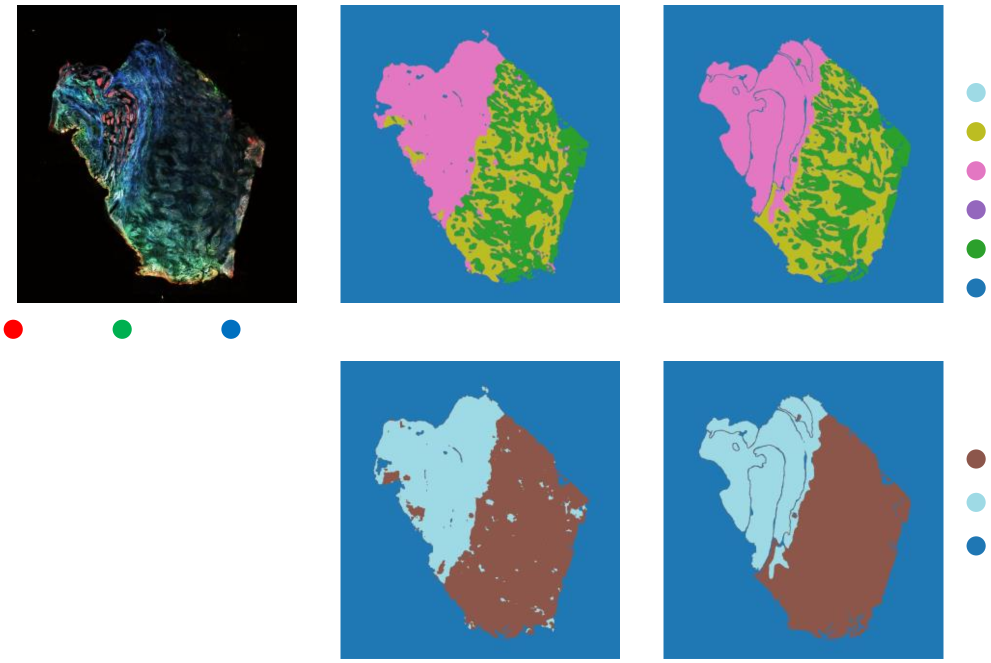

Fig. S11.
Model performance for sample 09.

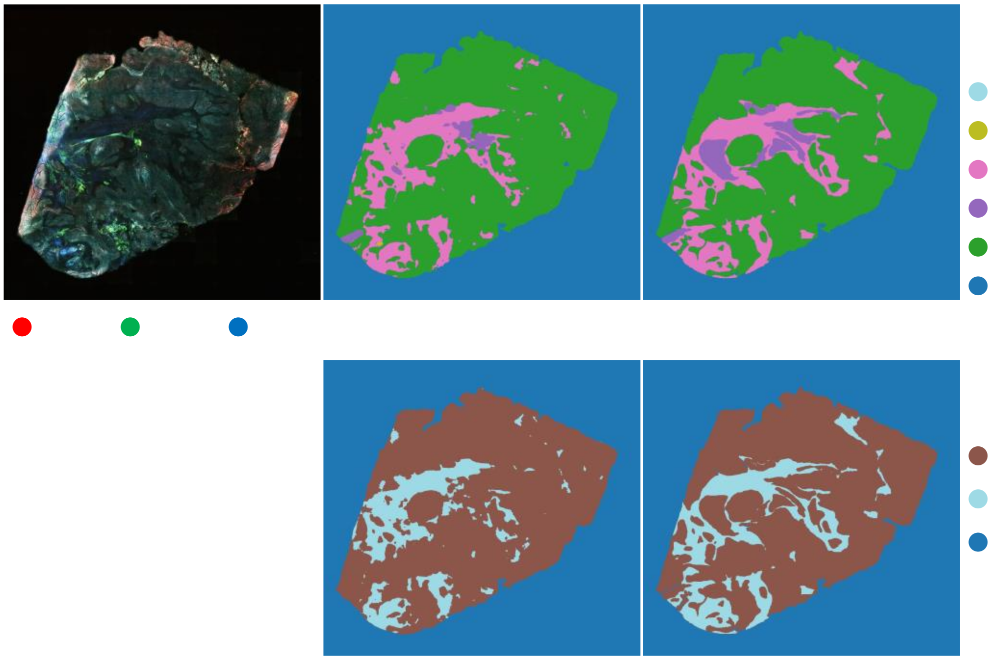

Fig. S12.
Model performance for sample 10.

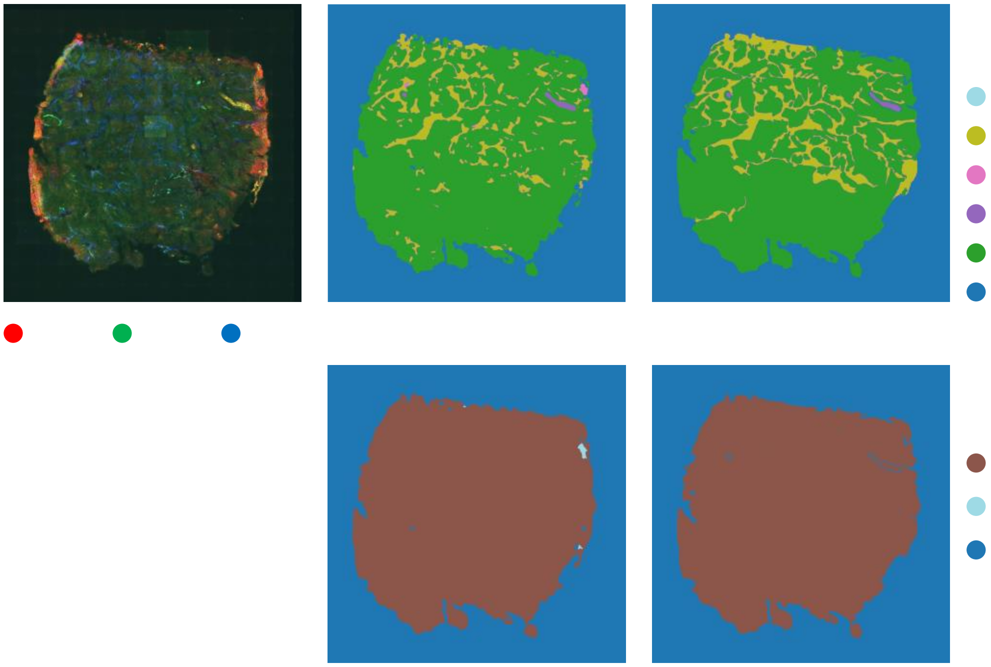

Fig. S13.
Model performance for sample 11.

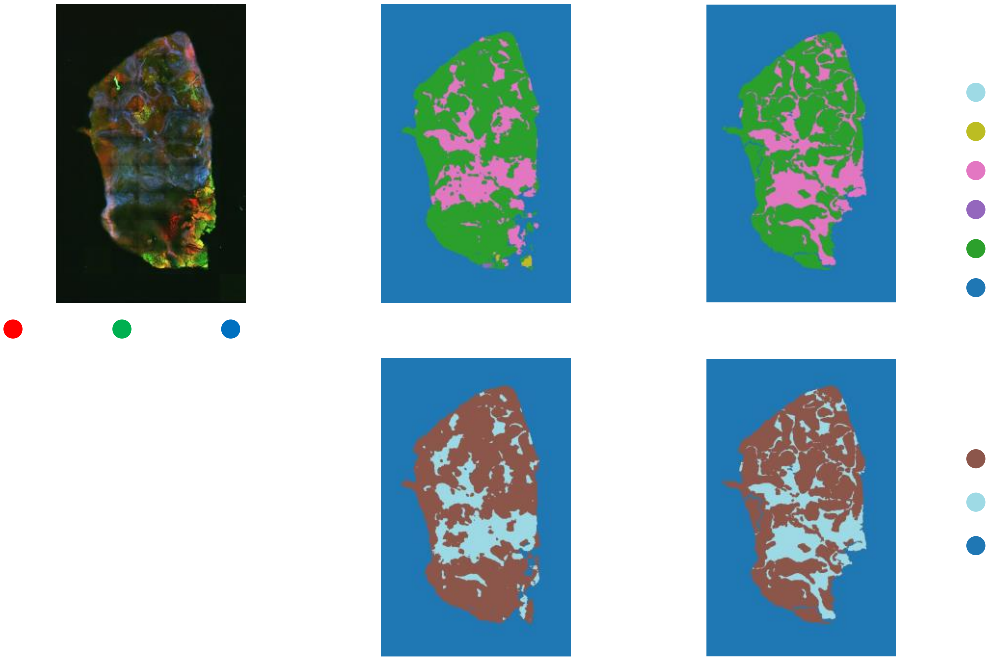

Fig. S14.
Model performance for sample 12.

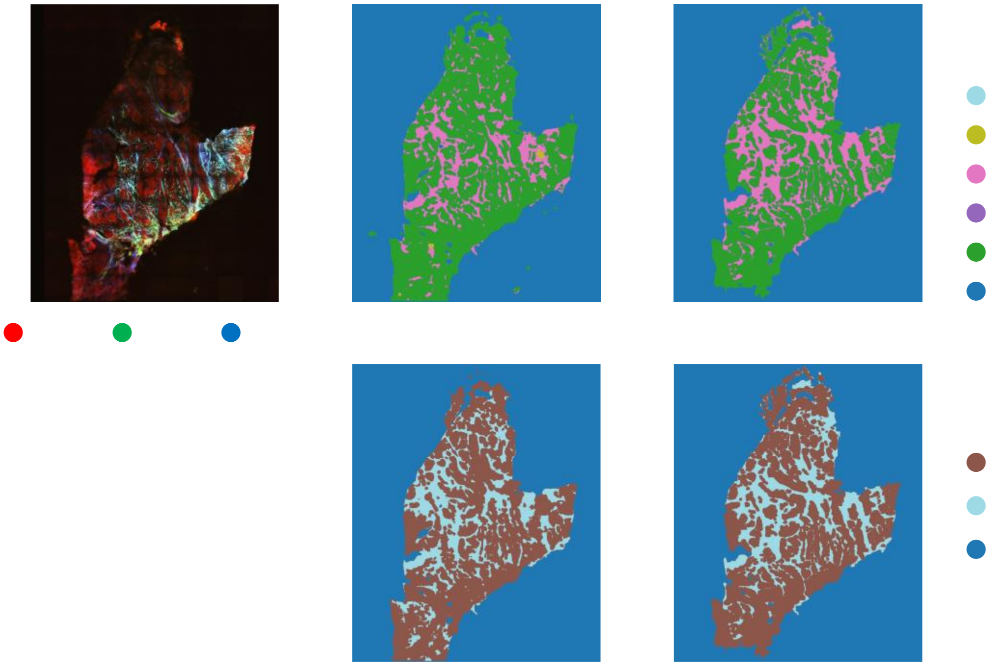

Fig. S15.

Model performance for sample 13.

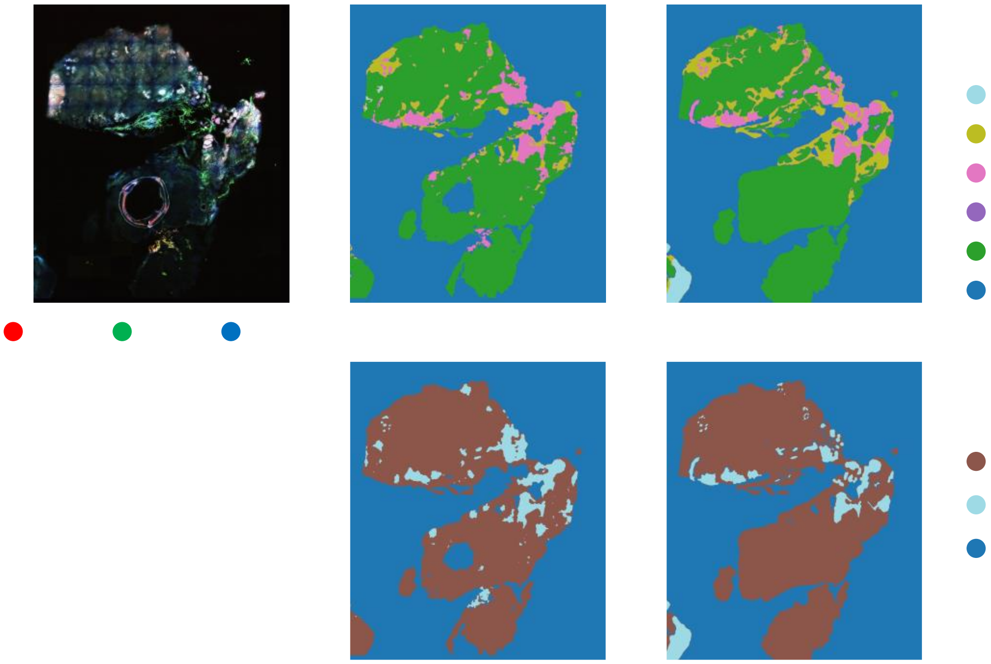

Fig. S16.

Model performance for sample 14-part1.

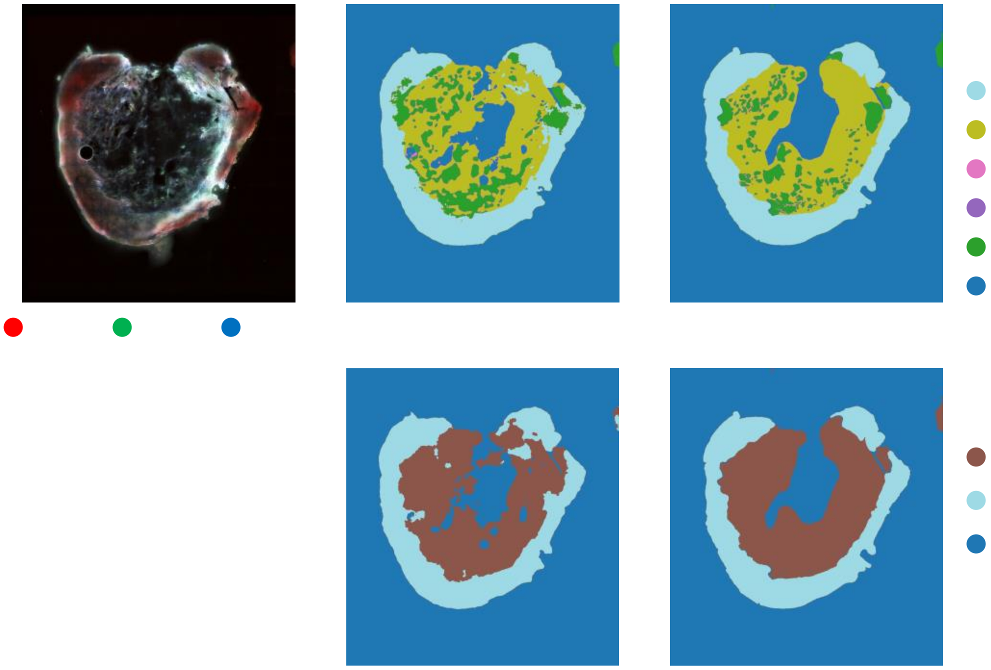

Fig. S17.
Model performance for sample 14-part2.

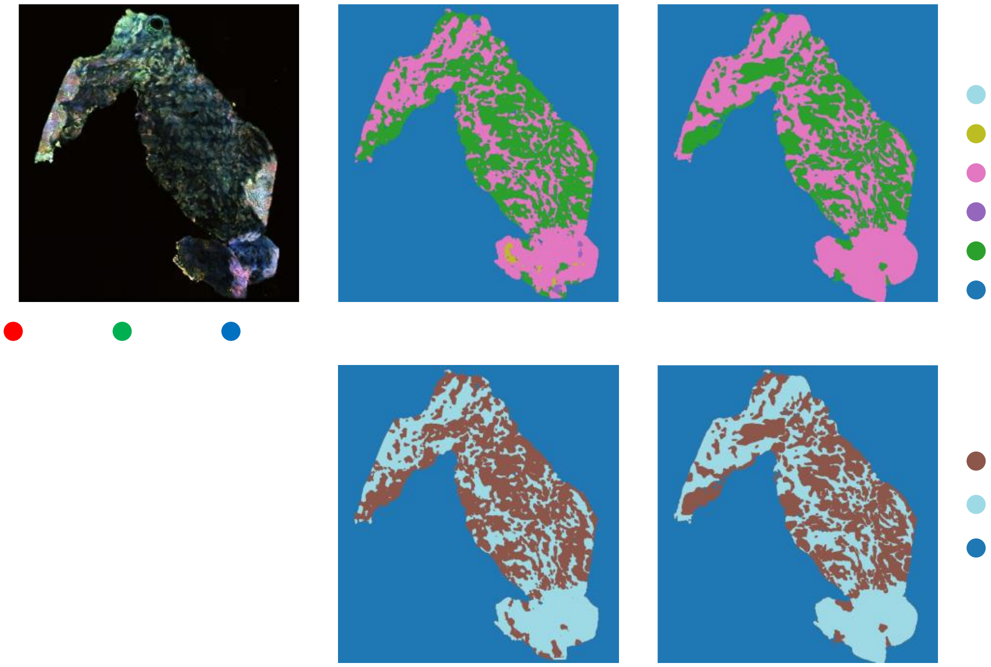

Fig. S18.

Model performance for sample 15.

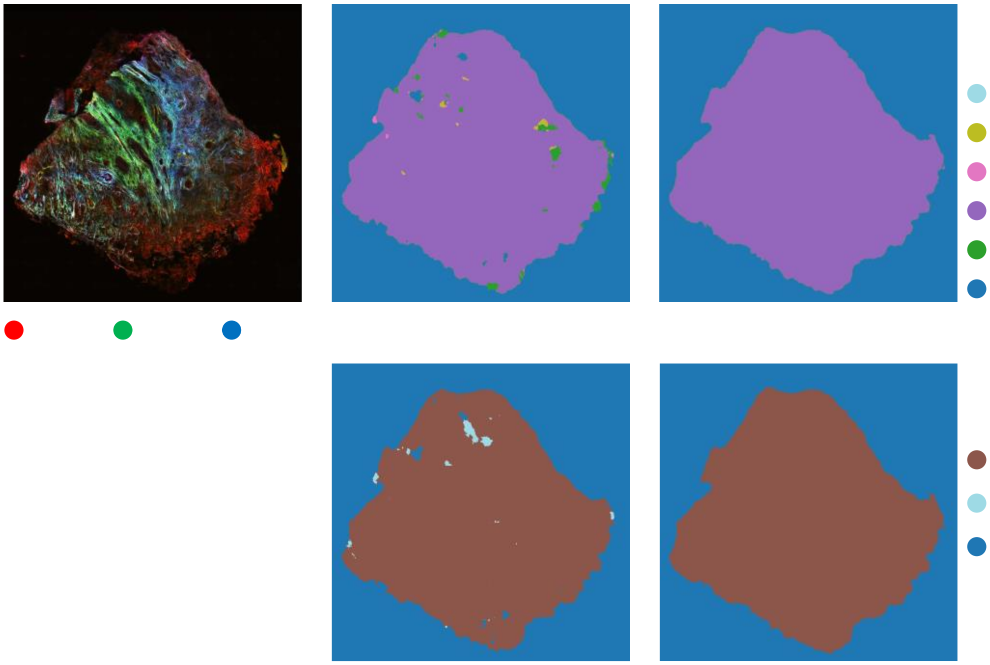

Fig. S19.

Model performance for sample 16.

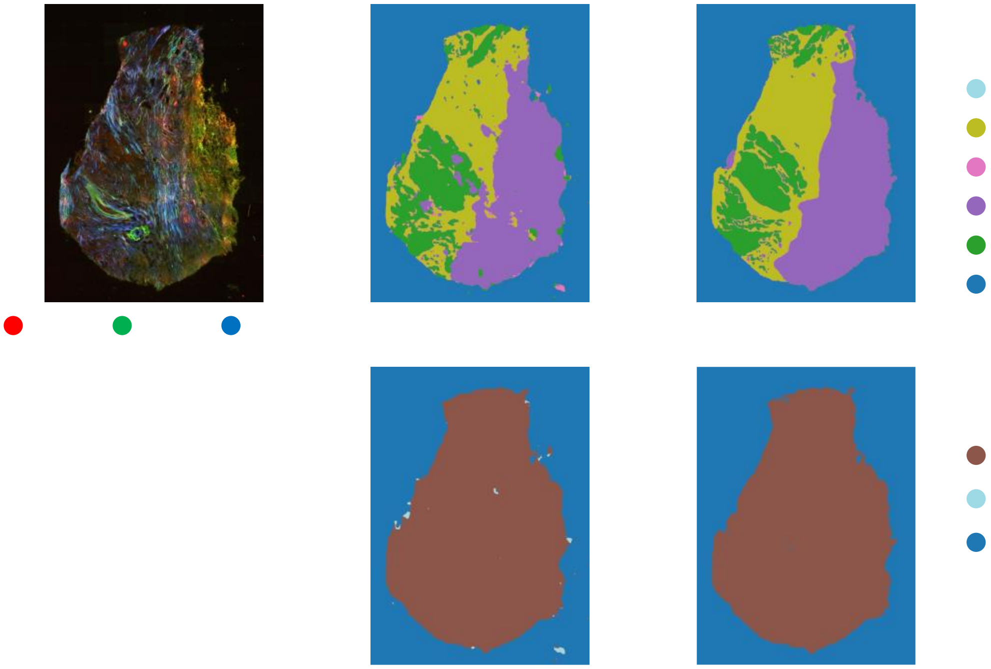

Fig. S20.
Model performance for sample 17.

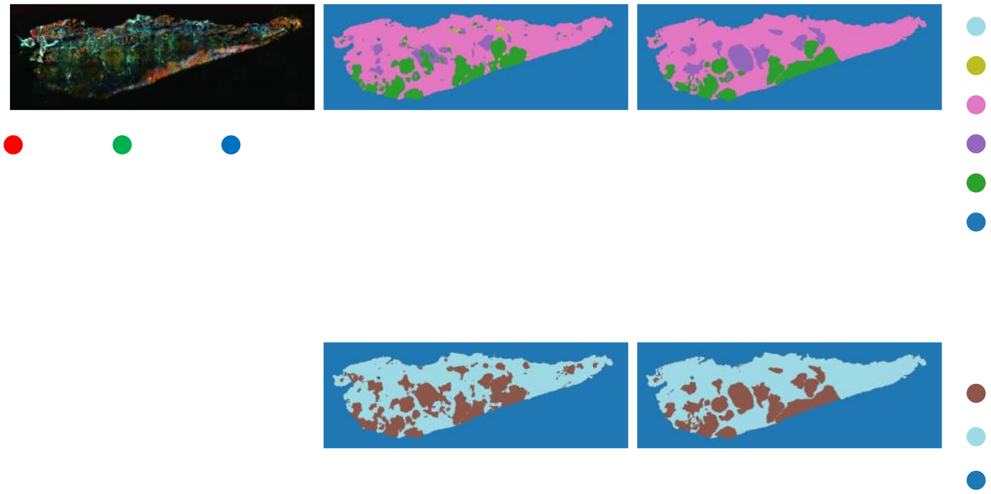

Fig. S21.
Model performance for sample 18.

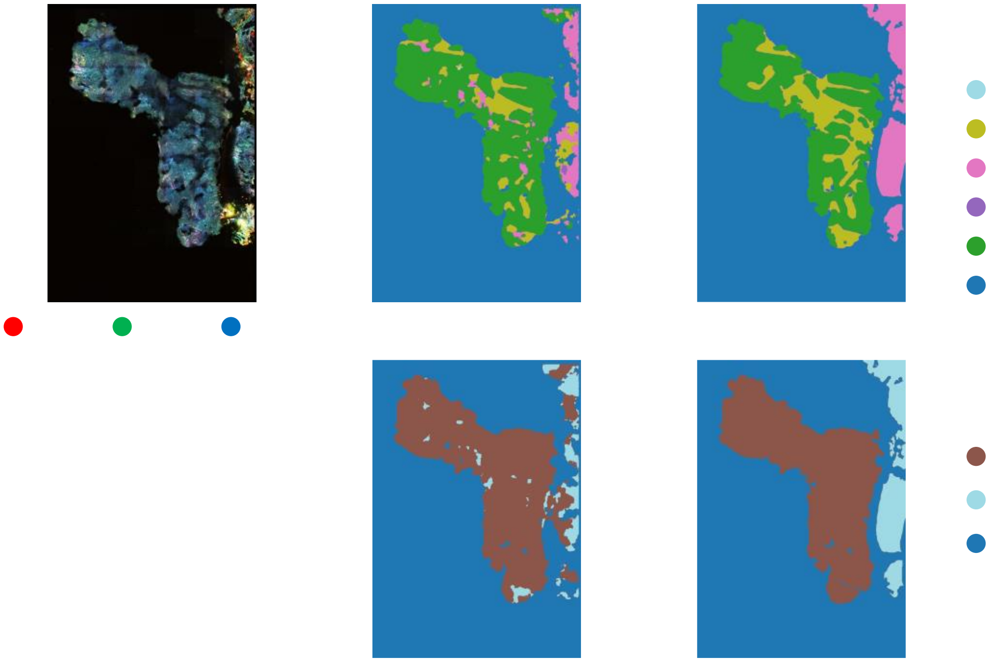

Fig. S22.

Model performance for sample 19-part1.

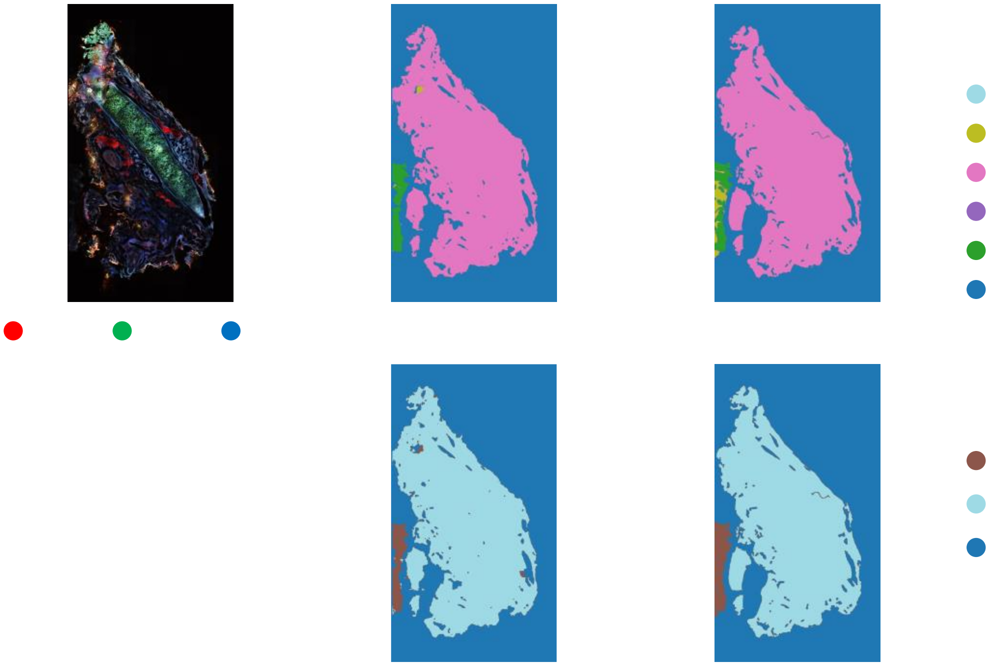

Fig. S23.
Model performance for sample 19-part2.

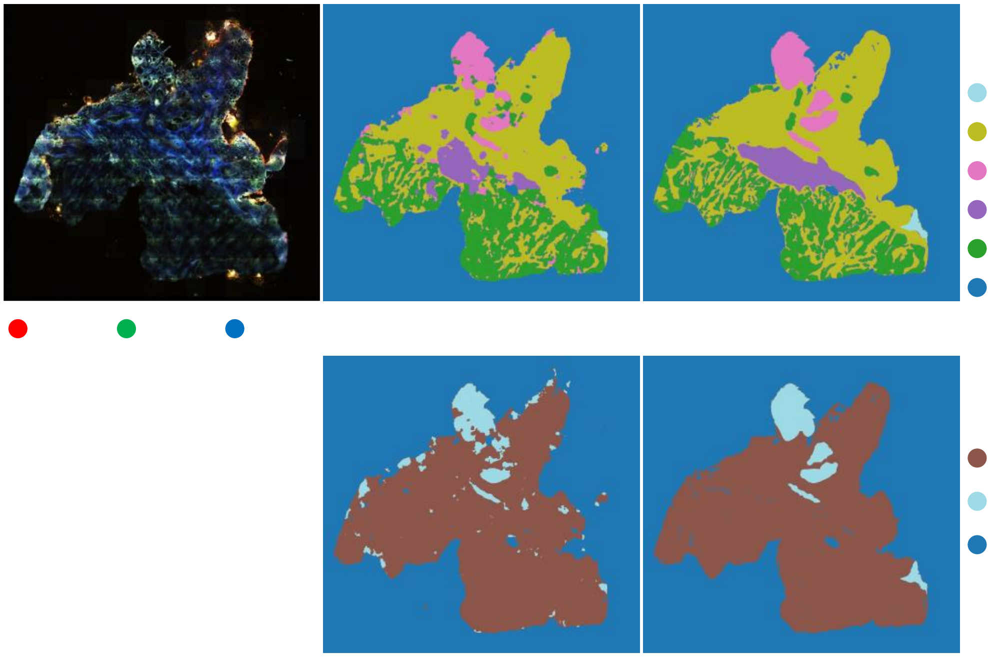

Fig. S24.

Model performance for sample 20.

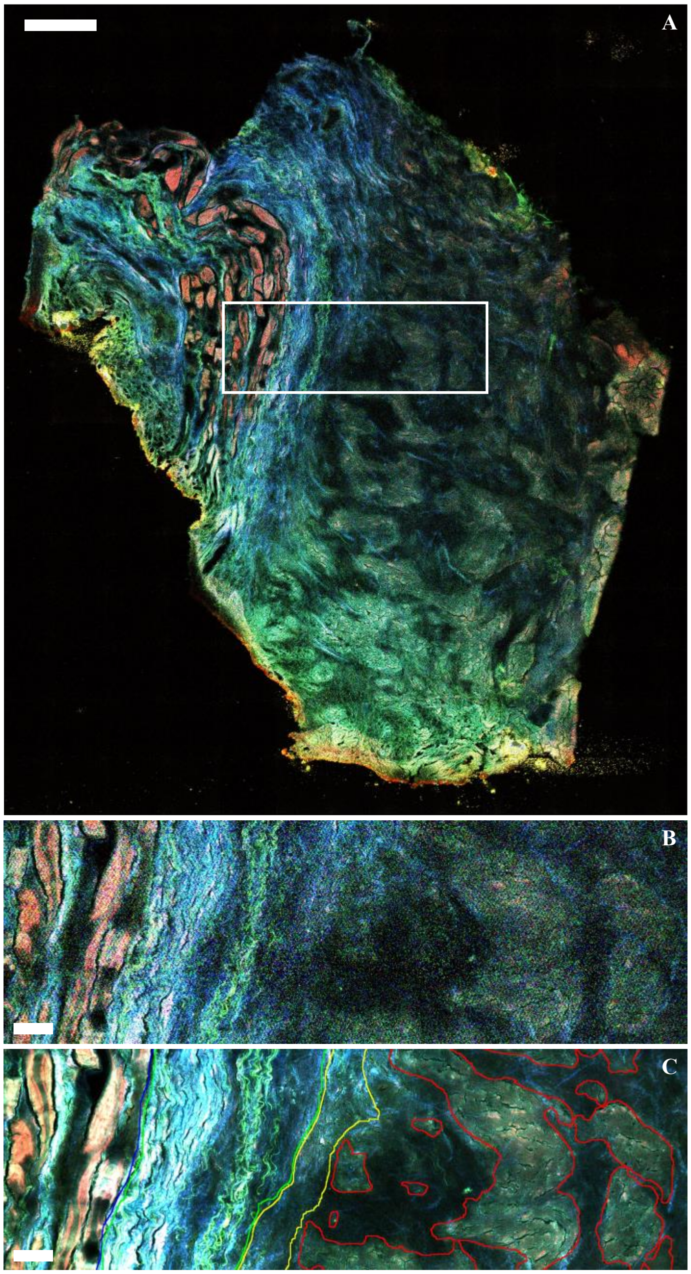

Fig. S25.
Low resolution fast imaging of the same sample shown in Fig. 1. (A) Overview image of the sample Scalebar is 0.5 mm. (B) Cropped area corresponding to the rectangular selection in (A). Scalebar is 100 µm. (C) Same cropped area from the high-resolution image (see Fig. 1 (C)). Scalebar is 100 µm. The low-resolution image is acquired with 250x250 pixels per tile, 3 µs pixel dwell time and no averaging, which corresponds to a frame rate of more than 3 frames per second (including a dead time between frames). Even in the case of fast scanning, good image quality is achieved, and the same tissue features can be distinguished. Only stitching and uneven illumination correction were performed, no further enhancements are applied.

Supplementary Text

Alternative uneven illumination correction

All the multimodal images were pre-processed and corrected for uneven illumination following the same workflow for the sake of uniformity. Since residual artifact can be observed in few images, we provide alternative corrections in Figs. S26, S27 and S28 and the relative predictions. The corrections are computed in the Fourier domain *(46)* for the samples displayed in Figs. S15 (Sample 13), S16 (Sample 14-part 1) and S24 (Sample 20), that show indeed a residual uneven illumination grid when corrected by BaSiC. In addition, we also include the raw image and the corresponding prediction result obtained without any prior correction of uneven illumination.

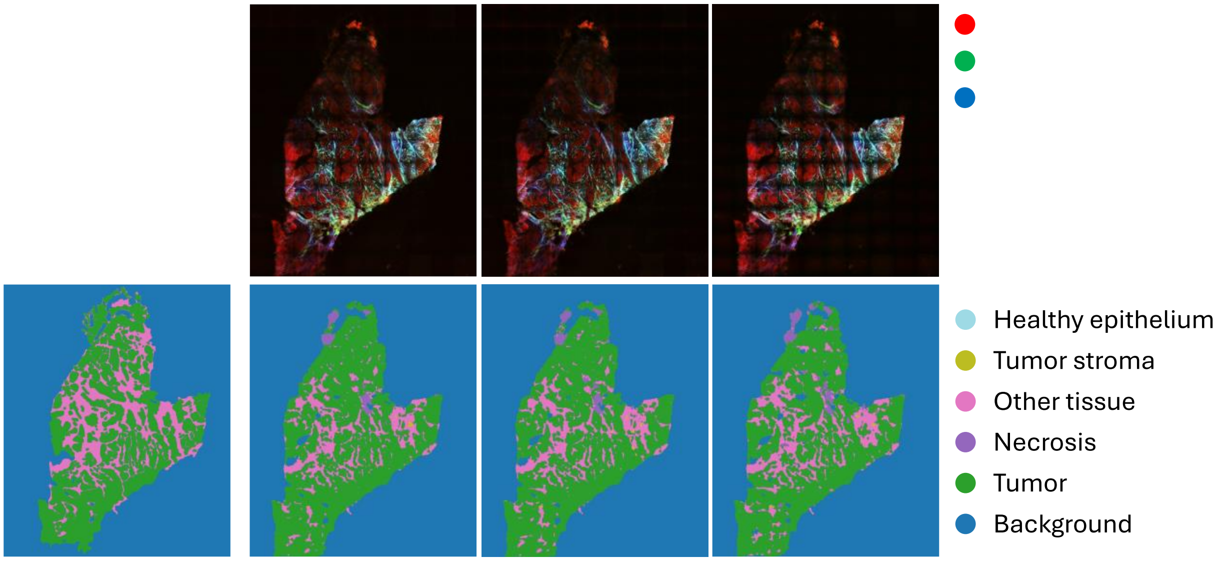

Fig. S26.
Model prediction for the raw image and alternative corrections of sample 13.

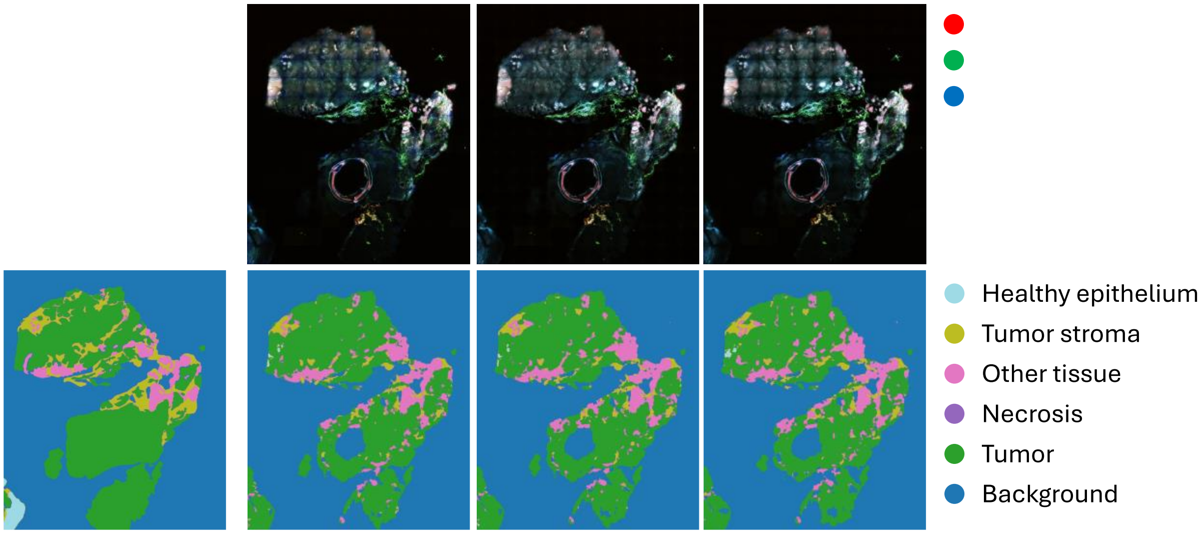

Fig. S27
Model prediction for the raw image and alternative corrections of sample 14-part1.

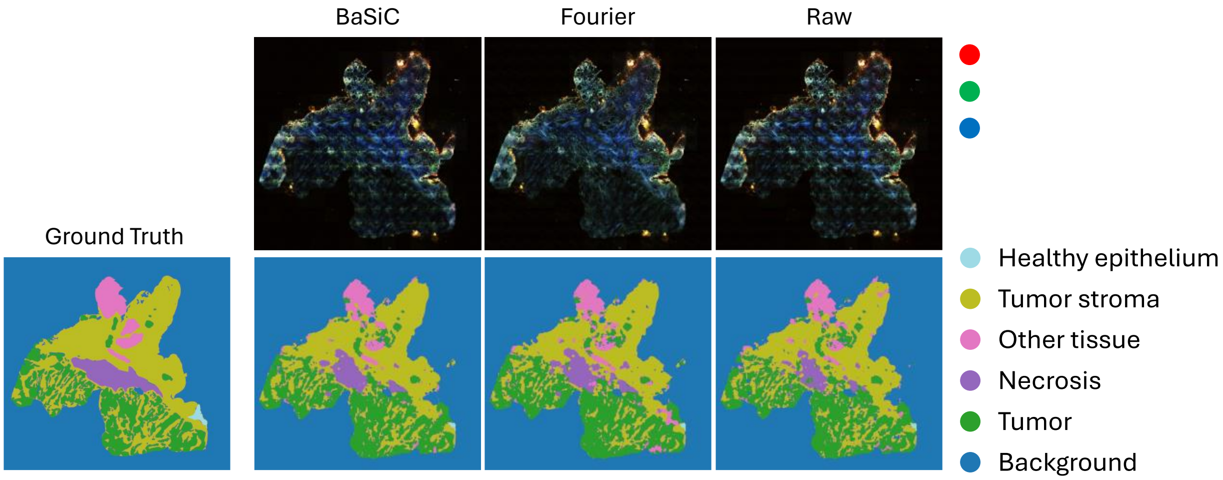

Fig. S28

Model prediction for the raw image and alternative corrections of sample 20.

Supplementary Text

Endomicroscope – Microsope comparison

The following figure S29 shows a comparison between the endomicroscope and a commercial laser scanning microscope (LSM) on the same tissue section, to demonstrate the reproducibility of the techniques. The images are acquired with same image scanning parameters: 1200x1200 pixels per tile, 3 µs pixel dwell time and 5 frames averaging.

The endomicroscope measurements were performed with the second prototype of the device, already introduced in the manuscript, with a pump and Stokes average power at the sample of 20 mW and 103 mW, respectively, at 5 MHz.

As for the LSM we used a state-of-the-art system (Stellaris DM8, Leica Microsystems GmbH) equipped with an OPO-based laser system (picoEmerald A.P.E.), which provides about 2 ps pump and Stokes pulses at 80 MHz repetition rate. The pump was set to 796.9 nm and the Stokes fixed at 1031.1 nm, thus probing the 2850 cm^{-1} Raman resonance. The average power was set to 20 mW for the pump and 102.9 mW for the Stokes, to match what used for the endomicroscope. CARS was acquired in the range 630-670 nm, SHG of the Stokes was detected between 513 and 518 nm and the TPEF channel collected all the signal below 495 nm. The detectors were set in photon counting mode, to measure the effective number of photons collected by the microscope. The objective was a 10x/0.4NA (HC PL APO CS2 DRY).

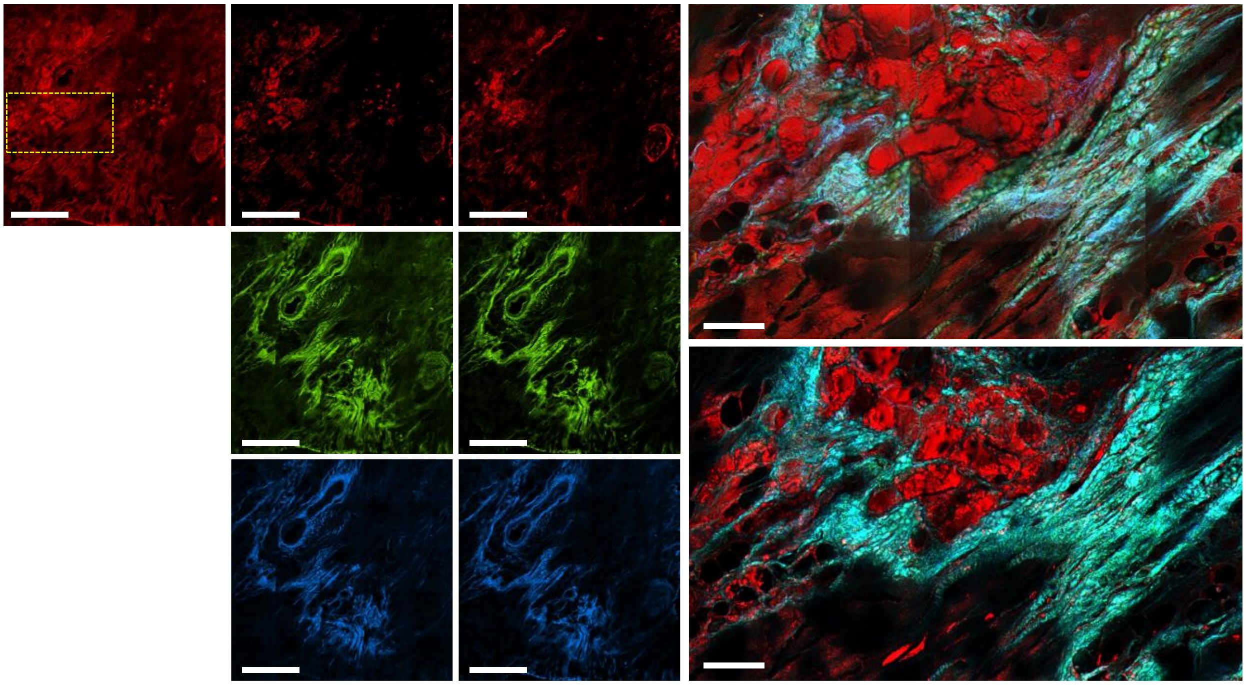

Fig. S29.
Visual comparison of the same region of interest (ROI) in the same tissue section measured with the endomicroscope and subsequently with a commercial laser scanning microscope (LSM).
As can be seen from the comparison, the individual channels appear very similar and exhibit comparable structures in all three channels. The CARS channels show better similarity when contrast adjustment is performed and lower intensity values are removed (see Fig. S29 (A1), S29 (A) and S29 (B)). This is probably due to the difference in spectral resolution among the instruments. The high resolution of the OPO-based system allows more specific signal generation and thus better contrast, whereas in our endoscopic device the non-resonant background is more prevalent. The TPEF and SHG channels (S29 (C-D) and S 29 (E-F), respectively) show a much better correspondence. Fig. S29 (G-H) depicts the merged RGB images of a sub-region of the ROI, showing good accordance in terms of visual appearance of the tissue structure. Scalebar is 0.5 mm in S29 (A1 to F) and 100 µm in S29 (G-H).

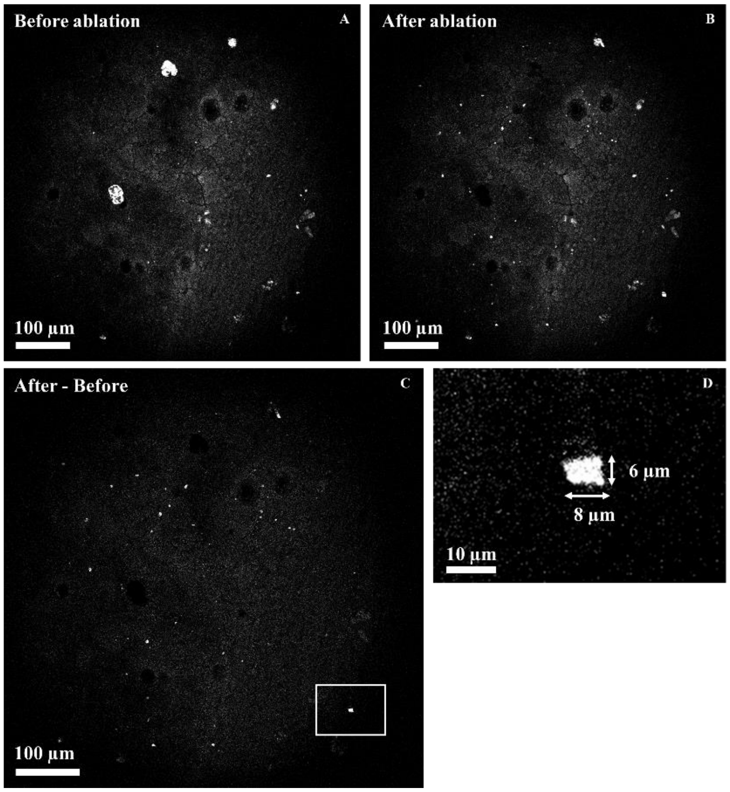

Fig. S30
Analysis of debris generated by ablation of cholesterol crystals. (A) SHG channel before ablation. (B) SHG channel after ablation. (C) Difference image between (B) and (A). (D) Zoom inset on the largest, manually identified particle, with a size of about ~ 6 µm x 8 µm.

Supplementary Text

Leave-one-out cross-validation

The following figures show the prediction results obtained during leave-one-out cross-validation for some selected samples. Fig. S31 shows the predictions of samples 02-part2 and 04, with poor segmentation performance. Fig. S32 shows the predictions of samples 07 and 09, for which the model shows an acceptable performance, by identifying the correct classes present in the tissue slices and highlighting the main tissue areas.

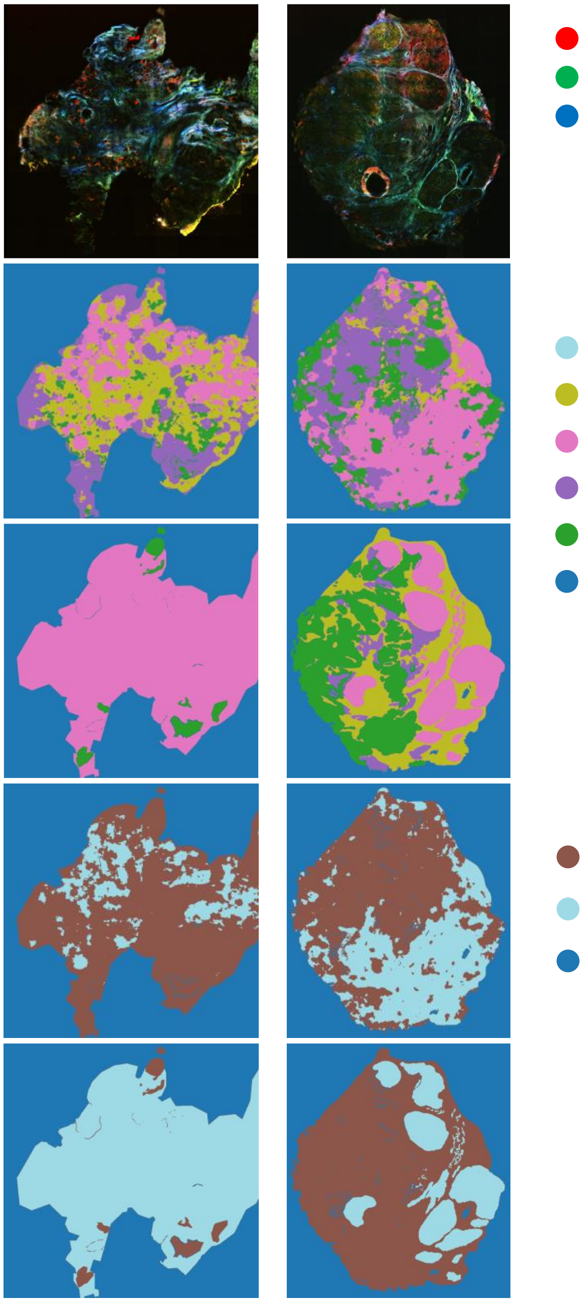

Fig. S31.
Model performance for samples 02-part2 (left column) and 04 (right column) using leave-one-out cross-validation: poor segmentation results.

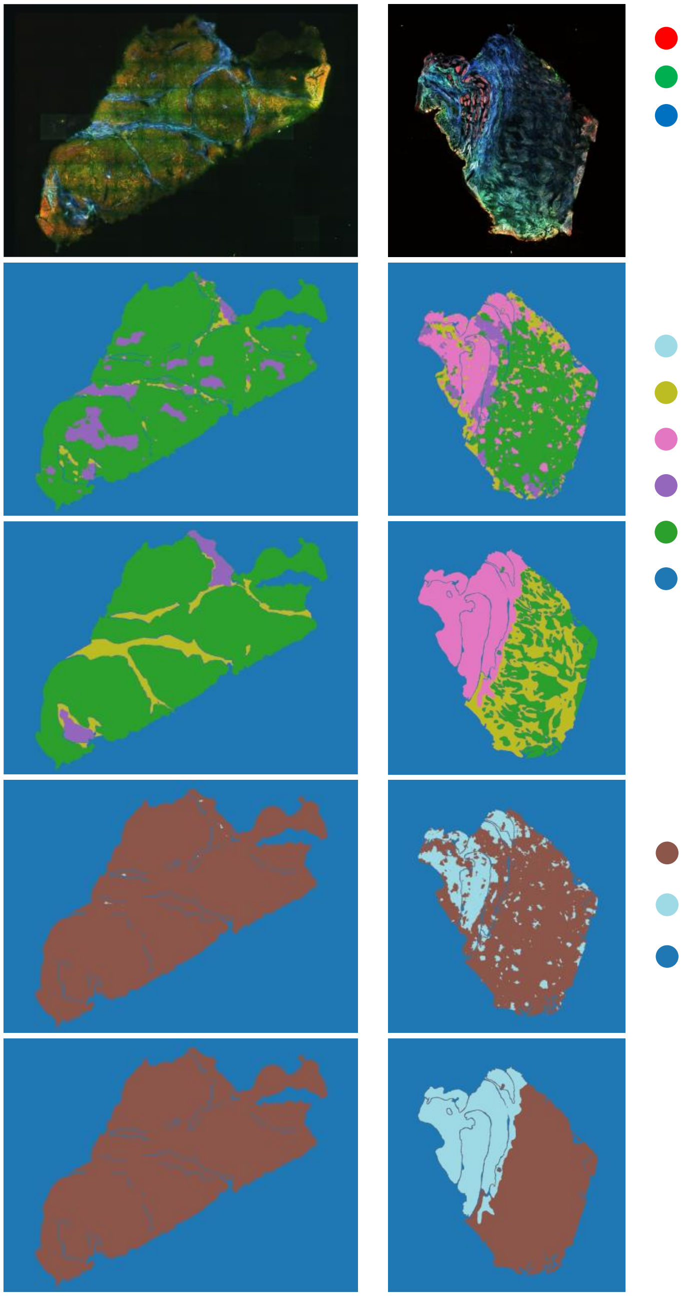

Fig S32.
Model performance for samples 07 (left column) and 09 (right column) using leave-one-out cross-validation: acceptable segmentation results.

Supplemetary Text

Samples biological variability

The following table reports detailed data about the measured samples and the biological variability of the patients. The right section of the table contains the patient number and the related samples measured from each patient, with the same nomenclature utilized in the manuscript and Supplementary Materials. For each sample, we also provide the operation date and the date in which the tissue slices have been created. The left section of the table contains, from left to right, the age of the patient at the operation date, the gender of the patient, and the original location (tumor localization and subsite) of the sample extracted during the surgical operation. The cross-validation approaches have been implemented by taking into consideration the dataset composition and by ensuring the correct separation into train and test split.

Sample data						Patient data			
**Patient**	**Measured samples**			**OP date**	**Tissue slices date**	**Age at op date in years**	**Gender**	**Tumor localization**	**Subsite**
patient 1	Sample 01			04.01.2018	08.12.2022	69	male		
patient 2	Sample 16			31.05.2018	20.03.2023	56	male	larynx	glottis
patient 3	Sample 18			05.07.2018	20.03.2023	68	male	oropharynx	base of tongue
patient 4	Sample 15	Sample 10	Sample 09	18.06.2019	07.03.2022	54	male	hypopharynx	
patient 4	Sample 19	Sample 20		18.06.2019	20.03.2023	54	male	hypopharynx	
patient 5	Sample 17			04.07.2019	20.03.2023	61	male	hypopharynx	
patient 6	Sample 14			08.07.2019	07.03.2022	67	male	hypopharynx	
patient 7	Sample 05			23.07.2019	07.03.2022	61	male	hypopharynx	
patient 7	Sample 07			08.08.2019	07.03.2022	61	male	hypopharynx	
patient 8	Sample 11			16.09.2019	07.03.2022	64	male	larynx	epiglottis
patient 9	Sample 03			18.03.2020	07.03.2022	66	male	oral cavity	floor of mouth
patient 10	Sample 06			19.03.2020	07.03.2022	34	female	oral cavity	tongue/floor of mouth
patient 11	Sample 04			31.03.2020	07.03.2022	47	male	oral cavity	
patient 12	Sample 02			30.07.2020	08.12.2022	77	male	oral cavity	tongue
patient 13	Sample 08			29.04.2022	07.03.2022	60	male	oral cavity	floor of mouth
patient 14	Sample 12			25.01.2018	20.03.2023	53	male	oropharynx	tonsil
patient 15	Sample 13			29.11.2018	20.03.2023	58	male	larynx	glottis

Table S1.
Information about measured samples and biological variability of the patients.

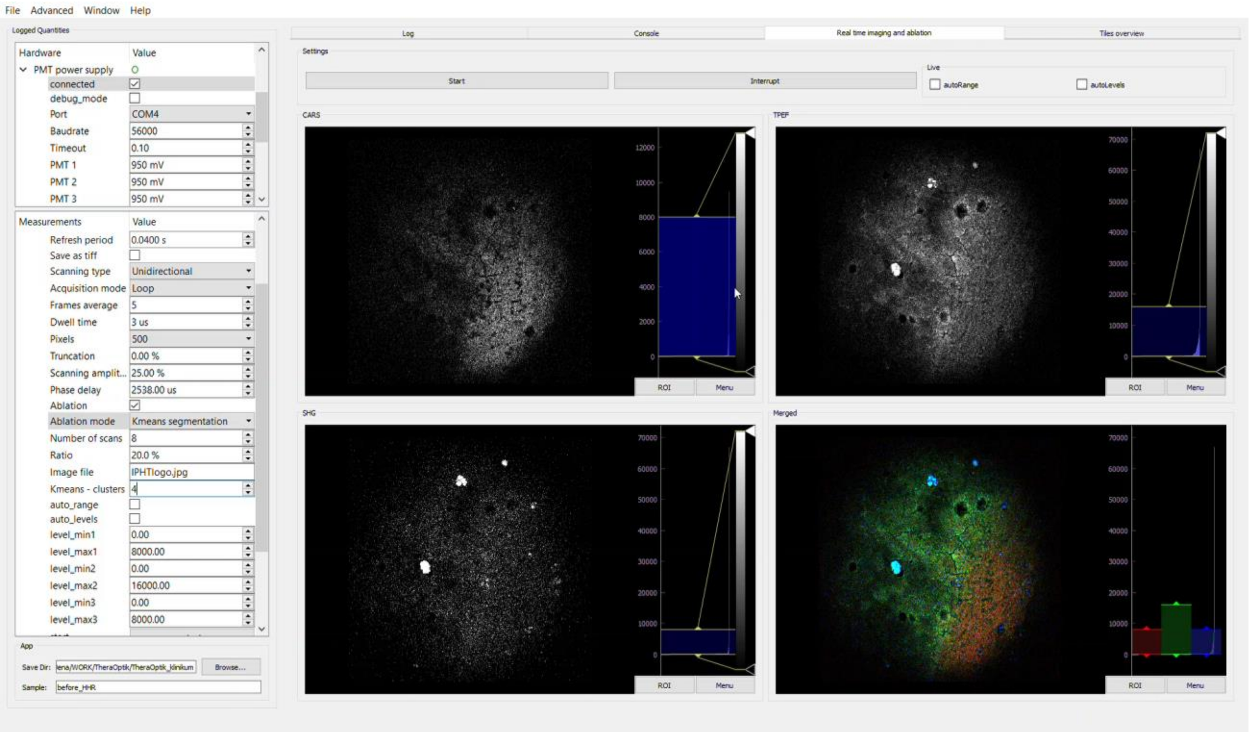

Movie S1.

Video of real-time device operation in the image processing-driven ablation workflow. Gray boxes indicate the current step in the pipeline. When the program starts, a first frame is acquired. Immediately after the acquisition, K-means clustering is performed on the image and the desired cluster is automatically chosen and converted to an ablation mask that is loaded on the DAQ card as voltage control to the AOM. Then, frame acquisition starts again and ablation is performed only in the desired area. The ablation is on for 8 consecutive frames in this example.
